# Modelling the impact of HIV and HCV prevention and treatment interventions for people who inject drugs in Dar es Salaam, Tanzania

**DOI:** 10.1002/jia2.25817

**Published:** 2021-10-18

**Authors:** Hannah Fraser, Jack Stone, Ernst Wisse, Veryeh Sambu, Peter Mfisi, Ivan J. Duran, Mireia Aguirre Soriano, Josephine G. Walker, Nobelrich Makere, Niklas Luhmann, William Kafura, Maieule Nouvellet, Allan Ragi, Bernard Mundia, Peter Vickerman

**Affiliations:** ^1^ Population HealthSciences Bristol Medical School University of Bristol Bristol UK; ^2^ Médecins duMonde Paris France; ^3^ National AIDS Control Programmes Dar es Salaam Tanzania; ^4^ The Drug Control and Enforcement Authority Prime Ministers Office Dar es Salaam Tanzania; ^5^ Tanzania Council for Social Development (TACOSODE) Dar es Salaam Tanzania; ^6^ Tanzania Commission for AIDS (TACAIDS) Dar es Salaam Tanzania; ^7^ Kenya AIDS NGO Consortium Nairobi Kenya

**Keywords:** hepatitis C virus, HIV, people who inject drugs, mathematical modelling, Dar es Salaam, Tanzania

## Abstract

**Introduction:**

People who inject drugs (PWID) in Dar es Salaam, Tanzania, have a high prevalence of HIV and hepatitis C virus (HCV). While needle and syringe programmes (NSP), opioid agonist therapy (OAT) and anti‐retroviral therapy (ART) are available in Tanzania, their coverage is sub‐optimal. We assess the impact of existing and scaled up harm reduction (HR) interventions on HIV and HCV transmission among PWID in Dar es Salaam.

**Methods:**

An HIV and HCV transmission model among PWID in Tanzania was calibrated to data over 2006–2018 on HIV (∼30% and ∼67% prevalence in males and females in 2011) and HCV prevalence (∼16% in 2017), numbers on HR interventions (5254 ever on OAT in 2018, 766–1479 accessing NSP in 2017) and ART coverage (63.1% in 2015). We evaluated the impact of existing interventions in 2019 and impact by 2030 of scaling‐up the coverage of OAT (to 50% of PWID), NSP (75%, both combined termed “full HR”) and ART (81% with 90% virally suppressed) from 2019, reducing sexual HIV transmission by 50%, and/or HCV‐treating 10% of PWID infected with HCV annually.

**Results:**

The model projects HIV and HCV prevalence of 19.0% (95% credibility interval: 16.4–21.2%) and 41.0% (24.4–49.0%) in 2019, respectively. For HIV, 24.6% (13.6–32.6%) and 70.3% (59.3–77.1%) of incident infections among male and female PWID are sexually transmitted, respectively. Due to their low coverage (22.8% for OAT, 16.3% for NSP in 2019), OAT and NSP averted 20.4% (12.9–24.7%) of HIV infections and 21.7% (17.0–25.2%) of HCV infections in 2019. Existing ART (68.5% coverage by 2019) averted 48.1% (29.7–64.3%) of HIV infections in 2019. Scaling up to full HR will reduce HIV and HCV incidence by 62.6% (52.5–74.0%) and 81.4% (56.7–81.4%), respectively, over 2019–2030; scaled up ART alongside full HR will decrease HIV incidence by 66.8% (55.6–77.5%), increasing to 81.5% (73.7–87.5%) when sexual risk is also reduced. HCV‐treatment alongside full HR will decrease HCV incidence by 92.4% (80.7–95.8%) by 2030.

**Conclusions:**

Combination interventions, including sexual risk reduction and HCV treatment, are needed to eliminate HCV and HIV among PWID in Tanzania.

## 1 INTRODUCTION

The adult prevalence of HIV in Tanzania was 4.7% in 2017 [1]. Estimates suggest that there are 30,000 people who inject drugs (PWID), with 30–50% living in Dar es Salaam [2]. HIV prevalence estimates vary among PWID in Dar es Salaam; decreasing from 35–51% over 2006–2011 [2, 3, 4] to 8.7–15.5% over 2014–2017 [5]. Female PWID have two‐fold greater HIV prevalence than males [3, 6], similar to elsewhere in sub‐Saharan Africa (SSA) [7, 8]. Uncertainty exists over the role of sexual risk behaviours to these HIV epidemics. The prevalence of hepatitis C virus (HCV) among PWID is lower in SSA than other regions [9], ranging from 16.2% to 30.3% [5, 6, 10] in Dar es Salaam.

Opioid agonist therapy (OAT) and needle and syringe programmes (NSP) can reduce the risk of HCV and HIV acquisition [11, 12, 13], but coverage remains low in SSA [14]. The first OAT clinic in Dar es Salaam started in 2011 [6, 15], with three clinics currently serving 5254 patients (Government data). NSP also started in 2011; however, there is only one fixed site with community outreach.

Anti‐retroviral therapy (ART) for people living with HIV (PLHIV) effectively reduces HIV transmission [16, 17, 18, 19, 20, 21]. ART has scaled up in Tanzania since 2010, with 75% of PLHIV on ART in 2019 [22] and 63.1% among PWID in 2015 [10, 23]. Although highly effective direct‐acting antiviral treatment exists for HCV [24], no PWID in Tanzania have been treated [10].

The World Health Organization (WHO) and UNAIDS have set goals for eliminating HIV and HCV by 2030 [25, 26], aimed at reducing HCV and HIV incidence by 90% by 2030. Although modelling has evaluated what interventions are required to achieve elimination [27, 28, 29, 30], no analyses have considered this for PWID in SSA. We developed the first model of HIV and HCV transmission among PWID in SSA. For Dar es Salaam, we determined the role of sexual and injecting HIV transmission among PWID and projected the impact of existing and scaled up interventions on the HIV and HCV epidemics.

## 2 METHODS

### 2.1 Model description

**Figure 1:**
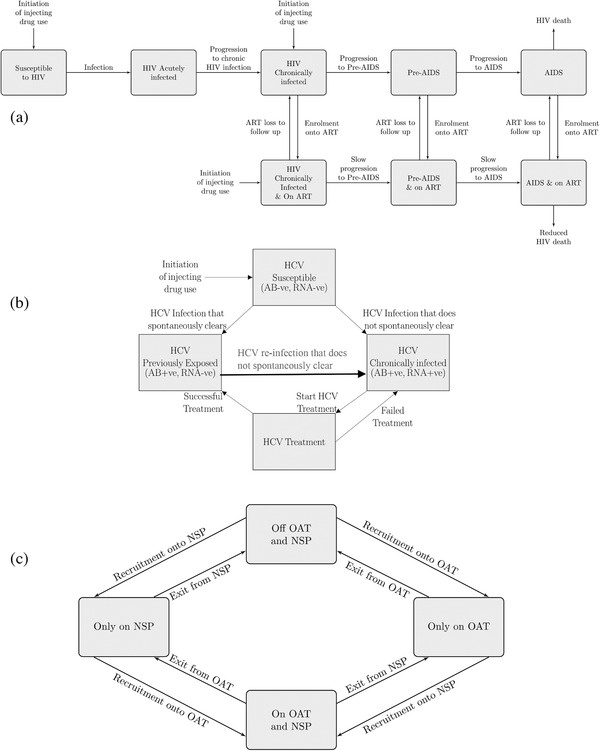
Model schematics for the different model stratifications. (a) Model schematic for HIV infection and ART status. Note that only individuals who are either chronically HIV infected, have pre‐AIDS or AIDS can be recruited on to ART. (b) Model schematic for HCV infection. (c) Model schematic for OAT and NSP coverage. Note that before scale‐up occurs in 2018, PWID can only be on OAT or NSP but not both. Abbreviations: AB, antibody; ART, anti‐retroviral therapy; HCV, hepatitis C virus; NSP, needle and syringe programmes; OAT, opioid agonist therapy; PWID, people who inject drugs; RNA, ribonucleic acid.

We developed a deterministic compartmental HIV and HCV transmission model among PWID, stratified by gender, HIV infection and treatment status, HCV infection and harm reduction (HR) state (Figure 1).

Individuals enter the model through initiating injecting drug use (IDU), susceptible to HCV, and not accessing OAT/NSP. A proportion are already infected with HIV. Entry is set to balance individuals leaving due to ceasing IDU and non‐HIV‐related death, but not HIV‐related mortality.

Susceptible PWID become infected with HIV and HCV through injecting‐related transmission, with HIV also sexually transmitted through contacts with PWID or non‐PWID. We assume that injecting‐related transmission does not vary by gender due to male and female PWID having similar injecting behaviour and HCV prevalences [31, 32] (Table S1). We only model heterosexual HIV transmission as few male PWID report sex with men (5.6% ever [31]). We assume that sexual HIV transmission differs by gender due to biological differences in risk [33], and large differences in HIV prevalence and sexual behaviours between male and female PWID (Table 1 and Table S2). HIV and HCV transmission risk also depends on the prevalence of infection in their sexual (just HIV) or injecting partners (HIV and HCV), with ART reducing sexual and injecting HIV transmission [16]. Injecting HIV and HCV transmission is also decreased for PWID on OAT and/or NSP [11, 12, 13]. We assume that PWID mix randomly to form transmission contacts with other PWID but vary this in a sensitivity analysis.

**Table 1:** Table showing data used to calibrate the model^a^

	Tanzania estimate	Date of estimate	Data source
Proportion of PWID who are female	3.5–18.2%	2013	Maximum [10] and minimum [6] across several estimates [15, 82, 83].
PWID population size	9000–15,000	2013	Consensus estimate of 30,000 (20,000–42,500) PWID in Tanzania, of whom 30–50% live in Dar es Salaam [2]. We take range of central estimate.
HIV prevalence among male PWID	27.6% (95% CI: 22.8–32.9%) 12.1% (95% CI: 7.9–17.5%) 44.9% (95% CI: 39.3–50.4%) 29.9% (95% CI: 24.0–36.2%) 14.8% (95% CI: 12.1–18.0%) 6.8% (95% CI: 4.9–9.1%)	Jan 2006 Jan 2010 Jan 2011 June 2011 2013 2017	[3] [46] [4] [6] [5] [5]
HIV prevalence among female PWID	63.9% (95% CI: 57.2–70.3%) 54.9% (95% CI: 44.2–65.4%) 71.4% (95% CI: 61.4–80.1%) 66.7% (95% CI: 49.0–81.4%) 25.5% (95% CI: 12.5–43.3%) 42.1% (95% CI: 24.6–59.3%)	Jan 2006 Jan 2010 Jan 2011 June 2011 2013 2017	[3] [46] [4] [6] [5] [5]
ART coverage among HIV‐positive PWID	34.8% (95% CI: 29.1–40.9%) 70.5% (95% CI: 44.0–89.7%) 63.1% (95% CI: 50.2–74.7%)	June 2011 Jan 2012 May 2015	[6, 31] [47] [10, 23]
HCV antibody prevalence among PWID	13.0–34.0%	June 2013	Minimum and maximum of 95% CIs across: [6] 27.7% (95% CI 22.0–34.0%) in June 2011 [10] 30.3% (95% CI: 27.9–32.8%) (February 2011–March 2016) [5] 16.2% (95% CI: 13.0–20.1%) 2017
Number of PWID on NSP	766–1479	2017	Programme data. Range is minimum to maximum of number of PWID accessing community and outreach services between January 2016 and August 2018, adjusted for the overlap calculated on data from March to August 2018
Number of PWID ever enrolled onto OAT	2099 2750 3718 4818 5254	2014 2015 2016 2017 Aug 2018	Data from Peter Mfisi, Tanzania Drug Control and Enforcement Authority
Duration between first time had sex and first time injected			Estimates calculated from MdM survey [31]
Males Females	7.5 years (95% CI: 6.7–8.3 years) 7.9 years (95% CI: 6.0–9.9 years)	2011 2011	

^a^ Estimates are either range (a–b) or mean with 95% confidence interval (CI). Note that dates of estimate are given as the mid‐point of when the study or studies were conducted.

Note that when mean and 95% CI were available, log likelihoods were used in the model calibration process; when ranges were available, the mid‐point of the range was used as the target; however, if the parameter set produced a value within the range, the calibration was assumed to have been achieved.

Abbreviations: ART, anti‐retroviral therapy; CI, confidence interval; HCV, hepatitis C virus; IBBS, integrated bio‐behavioural surveillance; MdM, Medicins du Monde; NSP, needle and syringe programmes; OAT, opioid agonist therapy; PWID, people who inject drugs; RDS, respondent‐driven sampling.

Following HIV infection, individuals progress through the acute, chronic, pre‐AIDS and AIDS phases of infection. Individuals in the acute and pre‐AIDS phases of infection have heightened infectivity [34]. Individuals with AIDS only engage in injecting and sexual behaviour if on ART. Individuals leave AIDS due to HIV‐related mortality. PLHIV (except acute) can be enrolled onto ART, which extends their survival. PWID receiving ART can be lost‐to‐follow‐up (LTFU) and then re‐enrolled onto ART at the same rate as ART‐naïve PWID.

Following HCV infection, some PWID spontaneously clear infection (differs by HIV infection [35, 36]), with the remainder progressing to chronic infection. Most PWID who receive HCV treatment achieve a sustained viral response (effective cure) or otherwise fail treatment and return to the chronically infected compartment. Re‐treatment occurs at the same rate as primary treatment. HIV‐HCV co‐infected PWID are assumed to be more infectious than HCV mono‐infected PWID [37].

PWID initiate and leave OAT and NSP at specific rates. Currently, PWID cannot access both OAT and NSP simultaneously, due to restrictions in Tanzania. This assumption is relaxed in our intervention scale‐up scenarios. Being on OAT increases the uptake of ART and improves viral suppression [38].

### 2.2 Model parameterization and calibration

The model was calibrated to data from Dar es Salaam. This includes data from a Médecins du Monde (MdM) bio‐behavioural survey from 2010 and their NSP provision, starting late 2010 [31]. Data also came from Integrated Bio‐Behavioral Surveillance (IBBS) surveys undertaken in 2014 and 2017 [5], and from Government OAT clinics in Dar es Salaam [6, 10, 39, 40]. Table 2 gives details of the surveys.

**Table 2:** Details of the bio‐behavioural surveys used by the model

					HIV prevalence			
Location	Year	Sample size	Recruitment method	HCV prevalence	Overall	Male	Female	Study reference
Dar es Salaam	May 2005–September 2006	534	Targeted sampling and snowball	NA	42.5% (95% CI: 38.3‐46.8)	27.6% (95% CI: 22.8–32.9)	63.9% (95% CI: 57.2–70.3)	[3]
Dar es Salaam	November 2009–March 2010	298	Targeted sampling and snowball	NA	25.6% (95% CI: 20.7‐31.0)	12.1% (95% CI: 7.9–17.5)	54.9% (95% CI: 44.2–65.4)	[46]
Kinondoni district, Dar es Salaam	November 2010–April 2011	419	PWID attending community outreach services	NA	51.1% (95% CI: 46.2‐56.0)	44.9% (95% CI: 39.3–50.4)	71.4% (95% CI: 61.4–80.1)	[4]
Temeke district, Dar es Salaam	June 2011	267	Targeted sampling and snowball	27.7% (95% CI 22.0–34.0)	34.8% (95% CI 29.1–40.9)	29.9% (95% CI: 24.0–36.2)	66.7% (95% CI: 49.0–81.4)	[6, 31]
Muhimbili Hospital OST clinic, Dar es Salaam	February 2011–March 2016	1350	New registered attendees of OST clinic	30.3% (95% CI: 27.9–32.8)	NA	NA	NA	[10]
Dar es Salaam	October–December 2013	620	Respondent‐driven sampling with five seeds	NA	15.5% (95% CI 14.5–17.1)	14.8% (95% CI: 12.1–18.0)	25.5% (95% CI: 12.5–43.3)	[5]
Dar es Salaam	November–December 2017	611	Respondent‐driven sampling with five seeds	16.2% (95% CI: 13.0–20.1)	8.7% (95% CI 6.4–11.8)	6.8% (95% CI: 4.9–9.1)	42.1% (95% CI: 24.6–59.3)	[5]

We assume that IDU initiated between 1998 and 2001 [41, 42], with 9000–15,000 [2] current PWID in Dar es Salaam. HIV and HCV were seeded among PWID at this time based on estimates of the HIV and HCV prevalence in the general population [43, 44]. ART is assumed to start in 2004 and scales‐up until 2015 (most recent data) whereupon coverage remains constant. The first OAT clinic started in Dar es Salaam in February 2011 with ∼3300 currently on OAT by August 2018 (Government data). NSP started in March 2011 with the number of people accessing the services increasing until April 2017. We assume the NSP reaches 753–1479 PWID each month over 2016–2018.

The level of sexual and injecting HIV transmission occurring among PWID was estimated stepwise. Levels of sexual HIV transmission were estimated by utilizing data from an MdM survey of PWID and PWUD in 2010. Logistic regression was used to estimate the HIV prevalence among PWID when they start injecting (0.3–5.3% for males and 1.3–45.4% for females in 2010). Then, using estimates of the duration that PWID had been sexually active before initiating injecting, we computed an average yearly risk of sexual HIV transmission over their pre‐injecting period. Because data from the 2010 MdM survey suggest that PWUD and PWID have similar sexual risk behaviours (Table S3), the same level of sexual HIV transmission risk was assumed during their injecting career. The rate of injecting HIV transmission was then calibrated to achieve the overall HIV prevalence among injectors (Table 1).

The model was calibrated using an approximate Bayesian computation Sequential Monte Carlo (ABC‐SMC) method [45] to the summary statistics in Table 1, including HIV and HCV prevalence estimates, intervention coverage estimates, PWID population size estimates and bounds for the levels of sexual HIV transmission [3, 4, 5, 10, 31, 46, 47]. Because initial attempts to fit the model could not reproduce the large decrease in HCV antibody prevalence estimates among all PWID (Table 1 [5, 6, 10]), one summary statistic for HCV prevalence was used in the ABC‐SMC method that accounted for the full uncertainty in the estimates (13.0–34.0%; applied in mid‐2013). Table 3 shows all parameter prior probability distributions. We conducted the ABC‐SMC multiple times, independently with random seeds, each time producing 5000 accepted parameter sets, until the median of key model projections converged (<5% difference from the previous combined sets; see Supporting Information). Convergence was achieved after seven implementations (Figure S2) giving 35,000 parameter sets. These were sampled (weighted by likelihood of their model fit) to give 3500 parameter sets, which were used to give the median and 95% credibility intervals (95% CrI; 2.5th to 97.5th percentile range) for all model projections. Further details are in the Supporting Information.

**Table 3:** Prior ranges for parameters used in the model

Parameter	Prior parameter distribution	Source
PWID‐related parameters		
Non‐HIV mortality rate among PWID (/year)	Normal: 3.53% (95% CI: 2.81–4.24%)	[84] Used to calculate mortality among male and female PWID
Ratio of crude non‐HIV mortality rates in male versus female PWID	Normal: 1.32 (95% CI: 1.21–1.44)	[84] Used to calculate mortality among male and female PWID
Average duration of injecting before cessation (years)	Triangular: 8 (IQR: 3–14)	Data from 2017 IBBS among PWID
Proportion of HCV infections among HIV negatives that spontaneously clear	Uniform: 0.22–0.29	[36]
Proportion of HCV infections among HIV positives that spontaneously clear	Uniform: 0.12–0.19	[35]
Year injecting drug use started ($Yr_{inj})$	Uniform: 1998–2001	Based on the experiences of drug users in Dar es Salaam in 2003 [41, 42]
Number of male PWID in $Yr_{inj}$	Uniform: 8000–30,000	Uninformative prior
Relative number of female PWID in $Yr_{inj}$ compared to male PWID	Uniform: 0.05–0.4	Range assumed based on prior knowledge of proportion of PWID population who are male
HIV‐related parameters		
Average duration of the acute stage of HIV infection (months)	Triangular: 2.9 (95% CI: 1.23–6.0)	[34]
Average duration of the pre‐AIDs stage of HIV infection (months)	Triangular: 9.0 (95% CI: 4.8–14.0)	[34]
Time to AIDs from infection (years)	Triangular: 9.4 (IQR: 5.5–10.1)	[85] Used to calculate average duration of the chronic HIV stage
Time to death from AIDS (months)	Lognormal: 10.0 (95% CI: 6.79–12.7)	[34]
HIV prevalence among new male PWID in June 2011	Uniform: 0.3–5.3%	Used MdM survey to estimate the HIV prevalence among male PWID at start of injecting
HIV prevalence among male adults in Dar es Salaam in 2011/2012	Normal: 5.3% (95% CI 3.9–7.2)	[86] Tanzania HIV/AIDS and Malaria Indicator Survey 2012
HIV prevalence among new female PWID in June 2011	Uniform: 1.3–45.4%	Used MdM survey to estimate the HIV prevalence among female PWID at start of injecting
HIV prevalence among female adults in Dar es Salaam 2011/2012	Normal: 8.2% (95% CI 6.6–10.1)	[86]
Transmissibility in acute stage	Lognormal: 276 (95% CI: 131–509)	[34]
Transmissibility in chronic stage	Lognormal: 10.6 (95% CI: 7.61–13.3)	[34]
Transmissibility in pre‐AIDS stage	Lognormal: 76 (95% CI: 41.3–128.0)	[34]
HIV sexual and injecting transmission parameters		
HIV sexual transmission rate from males to females	Uniform: 0–0.5	Uninformative prior
HIV sexual transmission rate from females to males	Uniform: 0–0.5	Uninformative prior
Proportion of male PWID's partners who are PWID	Normal: 0.165 (95% CI: 0.103–0.246)	[31]
Proportion of female PWID's partners who are PWID	Normal: 0.467 (95% CI: 0.253–0.657)	[31]
HIV injecting transmission rate	Uniform: 0–0.5	Unknown, varied to calibrate model to HIV prevalence trends
ART‐related parameters		
Relative rate of HIV progression if on ART	Uniform: 0.20–0.30	[70]
Relative ART coverage among individuals initiating injecting versus established PWID	Uniform: 0.7–0.9	Assumption
ART enrolment rate (/year) over 2004–June 2011 June 2011–March 2015 March 2015–2018	Uniform: 0–0.1 Uniform: 0–0.8 Uniform: 0–0.8	Unknown, varied to calibrate the model to ART coverage in 2011 and 2015 and then stable after 2015
ART LTFU rate per year among the general population	Uniform: 0.13–0.52	PEPFAR Tanzania Operational Plan Report FY 2013 (26% of care and treatment service clients no longer reported in the system)
Relative risk of ART LTFU among PWID versus general population	Lognormal: 1.36 (95% CI: 1.22–1.52)	[87]
Relative effectiveness of ART on reducing the transmission risk for injecting compared to reducing sexual transmission risk	Uniform: 0.7–1	Assumption as less evidence of the effectiveness of ART on injecting transmission than sexual transmission
To calculate relative reduction in HIV transmissibility if on ART		
Percentage of PWID on ART who are virally supressed (<400 copies per millilitre)	Normal: 80.7% (95% CI 73.3–86.8%)	ART data for PWID from Ministry of Health and general population [88]
Mean log_10_ HIV viral load copies per millilitre if on ART and virally suppressed	Normal: 1.35 (95% CI 1.26–1.44)	Lower limit of detection of viral load tests (400 copies/ml) undertaken in Tanzania
Mean log_10_ HIV viral load copies per millilitre if on ART and not virally suppressed	Normal: 4.07 (95% CI 3.72–4.42)	ART data from Tanzania Ministry of Health
Mean log_10_ HIV viral load copies per millilitre if not on ART and not virally suppressed	Normal: 4.84 (95% CI 4.06–5.45)	[89]
Increase in HIV transmissibility per log_10_ increase in HIV viral load	Lognormal: 2.45 (95% CI 1.85–3.26)	[90]
HCV‐related parameters		
HCV prevalence seed in 1980	0.5%	[43]
HCV injecting transmission rate	Uniform: 0.0001–2	Unknown, varied to calibrate to prevalence estimates
Increase in HCV transmissibility if HIV positive	Uniform: 1–7	[17]
Sustained viral response rate following HCV treatment	85–95%	[91]
OAT/NSP‐related parameters		
OAT enrolment rate (/year) from Feb 2011 to Jan 2014 Jan 2014 to Jan 2018	Uniform: 0–0.1 Uniform: 0–0.1	Unknown, varied to calibrate model to OAT trends over time
OAT exit rate (/year)	Uniform: 0.29–1.18	[40] Attrition from OAT 58.37/100 pyrs (–50% to +200%) in Dar es Salaam OAT clinic
Adjusted hazard ratio for attrition from OAT in females compared to males	Lognormal: 0.5 (95% CI: 0.28–0.89)	[40]
NSP enrolment rate (/year) from March 2011 to March 2017	Uniform: 0–1	Unknown, varied to calibrate model to NSP trends over time
NSP exit rate	Uniform: 0–6	Unknown, varied to calibrate model to NSP trends over time
Relative reduction in the risk of HCV transmission if on OAT	Lognormal: 0.50 (95% CI: 0.40–0.63)	[13]
Relative reduction in the risk of HCV transmission if on NSP	Lognormal: 0.44 (95% CI: 24–0.80)	[13]
Relative reduction in the risk of HIV transmission if on OAT	Lognormal: 0.46 (95% CI: 0.32–0.67)	[11]
Relative reduction in the risk of HIV transmission if on NSP	Lognormal: 0.42 (95% CI: 0.22–0.81)	[12]
Effect of OAT on percentage virally suppressed if on ART	1.45 (95% CI: 1.21–1.73)	[38]
Relative ART uptake rate if on OAT	1.87 (95% CI: 1.50–2.33)	[38]
Relative attrition rate from ART if on OAT	0.77 (95% CI: 0.66–0.95)	[38]

Abbreviations: ART, anti‐retroviral therapy; CI, confidence interval; HCV, hepatitis C virus; log10, logarithm to the base 10; MdM, Medicins du Monde; NSP, needle and syringe programmes; OAT, opioid agonist therapy; PWID, people who inject drugs; pyrs, person years.

Note that unless otherwise stated, triangular distributions are median (interquartile range) and normal distributions are mean (95% confidence interval).

### 2.3 Model analyses

We projected the status quo HIV and HCV epidemics among PWID until 2019 and estimated the percentage of HIV infections in 2019 and over 2019–2029 that were due to sexual transmission among male and female PWID. We then estimated the proportion of HIV and HCV infections that were prevented by OAT, NSP and/or ART in 2019.

We projected the percentage reduction in incidence of HIV and HCV over 2019–2030 if the coverage of OAT, NSP and ART were scaled up to 50%, 75% and 81% (90% of HIV‐positive PWID being diagnosed and 90% of diagnosed PWID on ART), respectively, with 90% of those on ART being virally supressed. These targets were chosen to represent the high coverage of OAT and NSP that some high‐income countries have achieved [48, 49] and align with WHO and UNAIDS targets for achieving HIV and HCV elimination. We then estimated the additional impact on HIV incidence of reducing sexual HIV transmission by 25%, 50% and 75%, in line with what has been achieved through sexual risk reduction interventions among drug users [50] and other male or high‐risk key populations [51, 52]. Lastly, we determined the impact of HCV‐treating 5%, 10% and 15% of infected PWID annually over 2019–2030, with or without joint scale‐up of NSP and OAT, to determine what is needed to achieve HCV elimination.

### 2.4 Uncertainty analysis

To determine which parameters are important for driving variability in our model projections, a linear regression analysis of covariance [53] was performed on the relative reduction in HIV and HCV incidence achieved over 2019–2030 from scaling‐up HR interventions and ART. The proportion of the model outcome's sum‐of‐squares contributed by each parameter was calculated to estimate the importance of individual parameters to the overall uncertainty.

As most of the data on ART coverage are from OAT patients, we also performed a sensitivity analysis considering the effect on our impact projections of assuming no further increase in ART coverage after 2011.

We also performed a sensitivity analysis considering the effect of including like‐with‐like mixing among PWID based on their contact with HR interventions on our impact projections of scaling‐up interventions. These analyses used existing model fits and either assumed 25% or 50% of PWID on OAT and/or NSP form injecting contacts with PWID on OAT and/or NSP (and same for PWID off OAT and NSP), with other contacts being randomly allocated.

## 3 RESULTS

### 3.1 Impact of existing interventions

**Figure 2:**
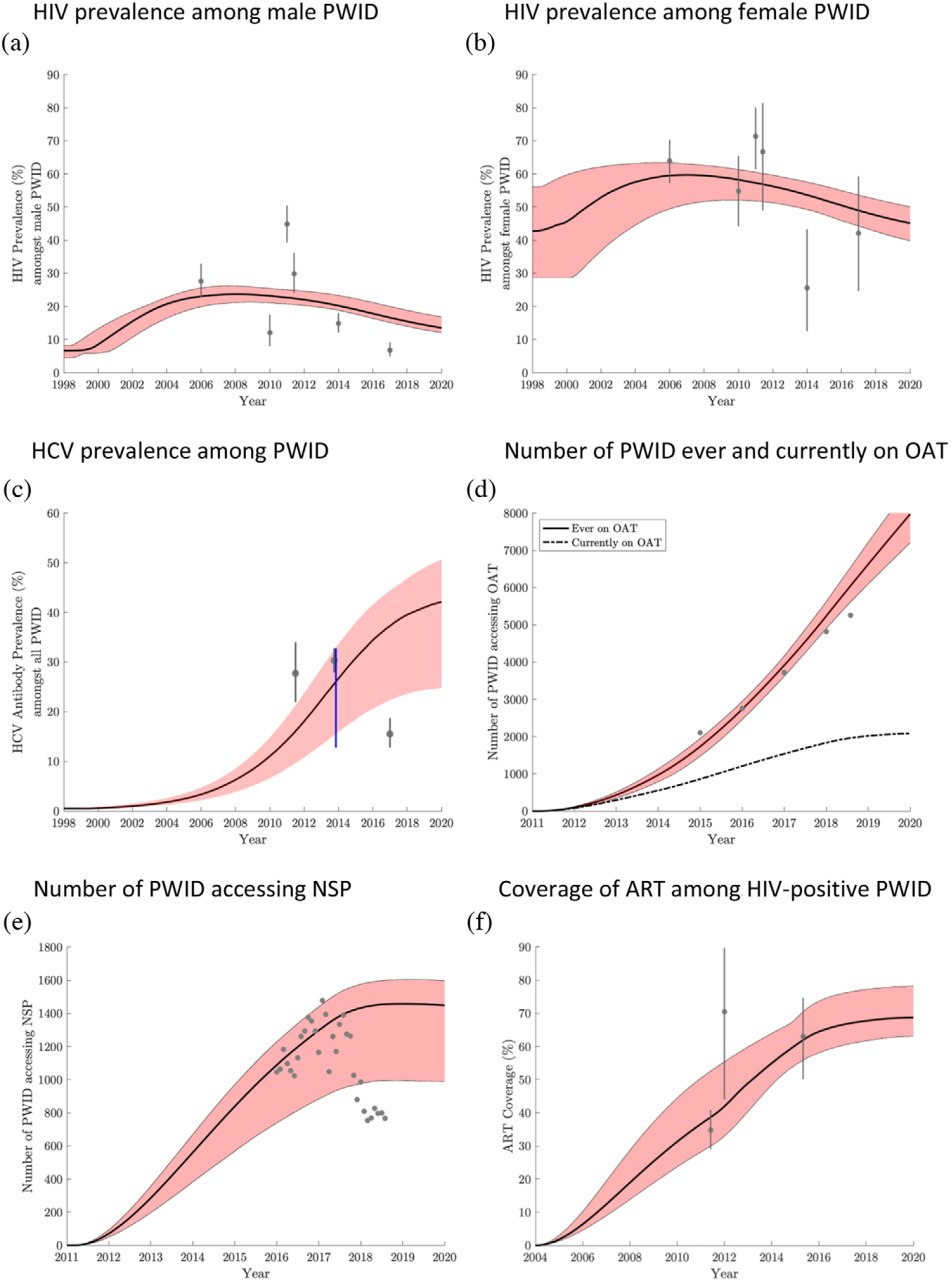
Model projections for the HIV prevalence among (a) male PWID and (b) female PWID over time; (c) HCV prevalence among all PWID; (d) number of PWID ever and currently on opioid agonist therapy (OAT); (e) number of PWID accessing needle and syringe programmes (NSP); and (f) coverage of ART among HIV‐positive PWID, including data estimates used to calibrate the model. For each, the black line gives the median projections from 3500 parameter sets, with 95% credibility intervals shown in red shading. Antiretroviral therapy started in 2004, OAT started in February 2011 and NSP started in March 2011. Grey circles and lines show the mean and 95% confidence interval of the data that the model was calibrated to, as given in Table 1. For (c), the blue line shows the range used for model calibration. Abbreviations: ART, anti‐retroviral therapy; HCV, hepatitis C virus; NSP, needle and syringe programmes; OAT, opioid agonist therapy; PWID, people who inject drugs.

The model agrees well with HIV and HCV prevalence data, with HIV prevalence slowly decreasing over 2004–2019, mainly due to increases in ART coverage (Figure 2). HIV prevalence is estimated to be three‐times higher among female PWID (46.2%, 95% CrI: 41.1–51.2%) than male PWID (14.4%, 95% CrI: 12.9–17.7%) in 2019, with projections suggesting that 70.3% (95% CrI: 59.3–77.1%) of new HIV infections in female PWID are due to sexual HIV transmission and 24.6% (95% CrI: 13.6–32.6%) in male PWID. The overall population attributable fraction due to sexual HIV transmission (removing transmission due to sexual transmission) over 2019–2029 is 56.4% (95% CrI: 39.8–66.9%). Model projections suggest the HCV epidemic is increasing until 2019, with an antibody prevalence of 41.0% (95% CrI: 24.4–49.0%) in 2019; similar across genders.

**Figure 3:**
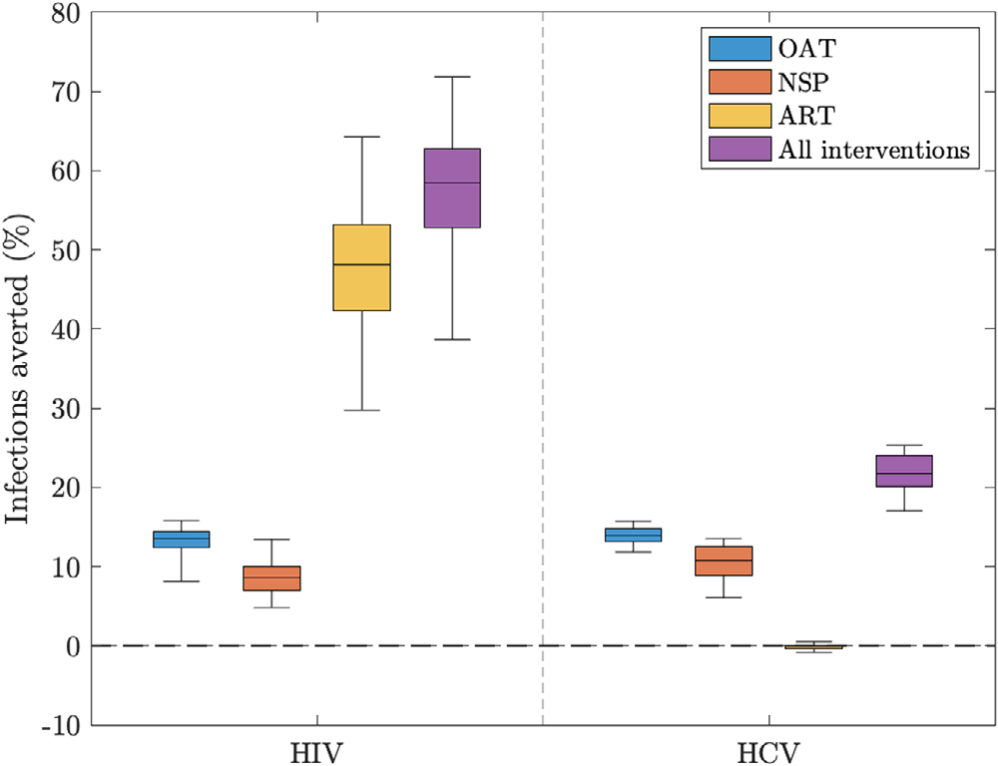
Box plot showing the percentage of HIV and HCV infections averted in 2019 by different interventions. Note that boxes represent the median and 25th to 75th percentile ranges and whiskers represent 2.5th and 97.5th percentiles. Antiretroviral therapy (ART) started in 2004; opioid agonist therapy (OAT) started in February 2011; and needle and syringe programmes (NSP) started in March 2011. Abbreviations: ART, anti‐retroviral therapy; HCV, hepatitis C virus; NSP, needle and syringe programmes; OAT, opioid agonist therapy; PWID, people who inject drugs.

The model projects that 22.8% (95% CrI: 18.7–29.6%) and 16.3% (95% CrI:11.1–18.5%) of PWID currently access OAT and NSP, respectively (Figure 2d, e). Due to this low coverage and a large role of sexual HIV transmission, OAT and NSP have had low impact so far, averting 13.5% (95% CrI: 8.1–15.8%) and 8.6% (95% CrI: 4.8–13.4%) of HIV infections, respectively, in 2019 (Figure 3) and 20.4% (95% CrI: 12.9–24.7%) combined. Conversely, the scale‐up of ART to 68.7% (95%CrI: 62.7–77.8%) coverage by 2019 (Figure 2f) averted 48.1% (95% CrI: 29.7–64.3%) of HIV infections in 2019. Combined, HR and ART prevented 58.5% (95% CrI: 38.7–71.9%) of HIV infections.

Existing OAT and NSP averted 13.9% (95% CrI: 11.8–15.7%) and 10.7% (95% CrI: 6.1–13.5%) of HCV infections in 2019, respectively, and 21.7% (95% CrI: 17.0–25.2%) combined.

### 3.2 Impact of scaling‐up interventions

**Figure 4:**
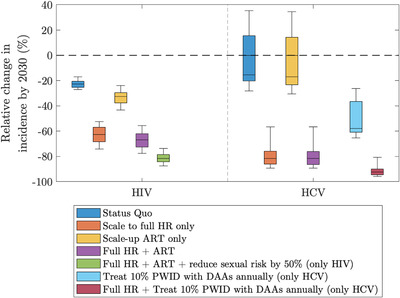
Box plot showing the relative change in HIV and HCV incidence among people who inject drugs (PWID) between 2019 and 2030 under different intervention scenarios. Note that boxes represent the median and 25th to 75th percentile ranges and whiskers represent 2.5th to 97.5th percentiles. Status quo is shown in darker blue shading, while intervention scenarios are (orange shading) scaling up opioid agonist therapy (OAT) to 50% coverage and needle and syringe programmes (NSP) to 75% coverage–denoted as full harm reduction (full HR); (yellow shading) scaling‐up antiretroviral therapy (ART) to 81% coverage with 90% of those on ART virally supressed; (purple shading) full HR and ART to high coverage; (green shading) full HR, ART to high coverage and decrease sexual risk by 50%; (pale blue shading) treat 10% of HCV‐infected PWID with direct‐acting antivirals (DAAs) annually; (brown shading) full HR and treat 10% of HCV‐infected PWID with DAAs annually. Abbreviations: ART, anti‐retroviral therapy; DAA, direct‐acting antiviral; HCV, hepatitis C virus; HR, harm reduction; NSP, needle and syringe programmes; OAT, opioid agonist therapy; PWID, people who inject drugs.

Figure 4 shows that scaling‐up OAT and NSP (denoted full HR) is projected to reduce the overall HIV incidence by 62.6% (95% CrI: 52.5–74.0%) from 2.2 (95% CrI: 1.1–3.2) in 2019 to 0.7 (95% CrI: 0.5–1.4) per 100 person years (/100 pyrs) by 2030 (Figure S9). Most (77.8%; 95% CrI: 61.7–87.5) of this impact is achieved through just scaling up NSP, with about half (46.7%; 95% CrI: 33.9–67.0) being achieved if only OAT is scaled up (Figure S8). Interestingly, similar impact is achieved from full HR among male and female PWID despite more HIV transmission being sexual among female PWID. This is due to the impact achieved among male PWID (Figure S3) also reducing levels of sexual HIV transmission among female PWID.

Due to the baseline ART coverage being high, scaling‐up ART alongside full HR interventions has little additional impact, reducing overall incidence by 66.8% (95% CrI: 55.6–77.5%) over 2019–2030. To achieve further impact by 2030, reducing sexual risk by 50% alongside full HR and ART reduces HIV incidence by 81.5% (95% CrI: 73.7–87.5%; Figures 4 and S4). Alternatively, if sexual risk is reduced by 25% or 75%, then HIV incidence decreases by 74.6% (95% CrI: 65.1–82.6) and 86.9% (95%CrI: 81.2–91.8) (Figure S4).

Continuing current levels of HR coverage, the model projects that HCV incidence will decrease from 14.1/100 pyr (95% CrI: 8.4–20.1) to 12.1/100 pyr (95% CrI: 7.0–21.4, Figure S10) by 2030, although a third (33.7%) of model projections suggest increasing HCV incidence and 61.0% suggest an increase in chronic prevalence. Scaling‐up to full HR results in an 81.4% (95%CrI: 56.7–89.2%) reduction in HCV incidence over 2019–2030 (Figure 4), reducing incidence to 2.6/100 pyr (95% CrI: 1.1–7.2) by 2030, with 86.7% (95% CrI: 70.0–93.3) of this impact being achieved if only NSP is scaled up and 47.8% (95% CrI: 22.3–60.0) if only OAT is scaled up (Figure S8). Treating 5%, 10% or 15% of HCV‐infected PWID per year with existing HR coverage results in a 39.8% (95% CrI:–1.1%to 49.3%), 57.9% (95%CrI: 26.3–65.3%) or 71.2% (95% CrI: 47.3–77.0%) reduction in HCV incidence by 2030, respectively (Figure 4 and Figure S6), with these increasing to 88.1% (95% CrI: 70.8–93.2%), 92.4% (95%CrI: 80.7–95.8%) or 95.2% (95% CrI: 87.3–97.4%), respectively, with full HR (Figure S6). The model suggests that these interventions will achieve less impact (Figure S11) if the baseline HCV incidence is still increasing over 2019–2030, although impact is still considerable with an 88.3% (95% CI: 79.0–93.9%) reduction in HCV incidence occurring if full HR is paired with 10% of HCV‐infected PWID being treated per year.

### 3.3 Uncertainty analysis

Analyses of covariance indicate that uncertainty in the sexual HIV transmission rate from females to males, the duration of injecting, the reduction in HIV transmission when on NSP and the recruitment rate onto ART after 2015 account for most (24.0%, 17.5%, 17.2% and 15.6%, respectively) variation in the percentage decrease in HIV incidence over 2019–2030 when HR and ART are scaled up. Conversely, variability in the projected decrease in HCV incidence over 2019–2030 is mainly driven by uncertainty in the reduction in HCV transmission risk when on NSP (54.3%) and the duration of IDU (38.3%).

If the rate of recruitment onto ART is assumed stable from 2011, the model projected ART coverage is 51.8% (95% CrI: 41.5–64.1%) instead of 68.7% (95% CrI: 62.7–77.8%) in 2019. This has minimal impact on HIV and HCV prevalence over time (Figure S7), with a slightly greater impact on incidence over time. ART is now projected to achieve less impact in 2019 (36.3% [95% CrI: 21.3–42.8%] infections averted). Incorporating like‐with‐like mixing has minimal effect on our existing model fits (Figures S12 and S13) or the impact (<1% difference) of scaling‐up prevention and treatment interventions (Tables S4 and S5).

## 4 DISCUSSION

The initiation of IDU in the late 1990s in Tanzania and other SSA countries has led to new sub‐epidemics of HIV and HCV in this region. Although both OAT and NSP were introduced in 2011 in Tanzania, there has been no evaluation of their prevention benefit among PWID in SSA. This is particularly an issue for HIV because there is likely to be high rates of sexual HIV transmission in these settings. Our modelling study helps fill this knowledge gap, projecting the impact of OAT, NSP, ART and HCV‐treatment on HIV and HCV incidence in Dar es Salaam, Tanzania. Our results indicate that due to their low coverage (OAT 22.8%; NSP 16.3% in 2019), OAT and NSP have had a small impact to date, only averting 20.4% and 21.7% of HIV and HCV infections among PWID, respectively, in 2019. Due to being at higher coverage (68.7%), ART has had greater impact, averting 48.1% of HIV infections in 2019.

For reducing HIV transmission going forward, increasing the coverage of HR interventions and ART among PWID to high levels will prevent two‐thirds of new HIV infections by 2030, but will be insufficient to reach the UNAIDS target of decreasing HIV incidence by 90%. To achieve this, large reductions (>75%) in sexual risk will also be needed. In contrast, HCV incidence can reduce by 81.4% by 2030 solely through increasing the coverage of OAT and NSP, with only 10% of HCV‐infected PWID needing treatment annually to achieve the WHO target of reducing HCV incidence by 90% by 2030. The large potential impact of HR interventions on HCV in this setting is due to the lower prevalence of infection in Tanzania (and SSA) than for other settings. This contrasts with previous modelling and data from other settings that has suggested HR interventions can or have impacted on HCV transmission, but to a more limited extent [54, 55, 56, 57, 58, 59, 60, 61].

### 4.1 Strengths and limitations

The strengths of our analyses are that we use multiple data sources to develop the first HIV‐HCV co‐infection transmission model for PWID in SSA. However, our analyses have limitations. Firstly, estimates of HIV prevalence varied widely and decreased between older and recent surveys. Data on the HCV epidemic were also limited, similar to most sub‐Saharan countries [62], and exhibited similar patterns of lower prevalence in the latest survey. It is uncertain if these variations are due to changes in the HIV and HCV epidemic or differences in the sampling methods used in different surveys (targeted and/or snowball sampling [3, 6, 46] vs. respondent‐driven sampling [5]) and associated biases. As more HIV and HCV prevalence estimates become available, we will get a better idea of the real trends in these epidemics and the precision of our projections will improve. However, by including all currently available data, our results are as robust as possible.

Secondly, although the effectiveness of OAT and NSP in reducing HIV [11, 12] and HCV transmission risks [13] were both estimated from global systematic reviews, most review data came from Europe, Australia and North America. It is uncertain whether these interventions would have similar effectiveness in Tanzania, highlighting a need for research to evaluate these interventions in lower income countries.

Thirdly, limited sexual behaviour data meant we modelled sexual HIV transmission simply. Our calibrated models fit observed HIV and HCV prevalence patterns well for male and female PWID, with available data suggesting substantial sexual HIV transmission because the estimated HIV prevalence among newly initiated PWID is quite high [31]. This is also supported by available HIV‐HCV co‐infection data, which suggests that many HIV‐positive PWID do not have HCV (48.4%) and so are likely to have been infected sexually [63]. We also did not have any data on how PWID mix to form injecting or sexual partnerships, and so random mixing was assumed. Previous modelling suggests that this should not have majorly affected our model projections [54, 64] as confirmed by our sensitivity analyses, except possibly for HCV treatment, where network modelling may suggest that less impact will be achieved [65]. Encouragingly, the realism of our model‐based impact projections for HCV treatment has recently been validated against empirical HCV incidence data [66], and so this should not be a concern. Despite this, network modelling would still be an important addition to understand whether incorporating details of who someone mixes with could also affect the impact and design of the interventions that are introduced.

Finally, the model does not account for any effects of the global coronavirus pandemic on intervention programming in 2020. This is because data suggest the effects were small, with OAT operating at similar levels (7425 enrolments for 2020), NSP continuing in Dar es Salaam and ART clinics remaining open. Unfortunately, though, the extent of the COVID‐19 epidemic in Tanzania is unknown (no reporting since May 2020) and so we do not know whether intervention activities may be affected in the future.

### 4.2 Comparison with existing literature

A wealth of HIV modelling exists for SSA, with numerous studies considering key populations [67, 68, 69, 70, 71]. However, to our knowledge, this study, along with a sister study considering Kenya (in preparation), presents the first modelling of HIV and HCV transmission among PWID in SSA. Only two previous studies have modelled HIV or HCV transmission among PWID in SSA, with one considering the potential impact of OAT on HIV in Kenya [67] and another estimating the impact and cost‐effectiveness of prevention and treatment strategies for HCV in Dar es Salaam [72]. A decision tree model has also been used to compare the cost‐effectiveness of difference strategies for diagnosing HCV in Senegal [73]. In contrast to other studies modelling the requirements for eliminating HCV among PWID, our findings suggest that scaling‐up OAT and NSP could nearly achieve the WHO HCV elimination targets in Tanzania by 2030, [37, 74] with only low levels of HCV‐treatment being needed. However, similar to other HIV modelling among PWID [75, 76], our results highlight the importance of combined interventions for decreasing HIV incidence among PWID, but with the major difference of a considerable focus also being needed on reducing sexual HIV transmission risks in Tanzania.

## 5 CONCLUSIONS

Tanzania and many other sub‐Saharan countries are witnessing problems of increased injecting drug use, with many having no HR intervention coverage [14, 77, 78]. While ART has been scaled up across the general population in Tanzania, additional scale‐up among PWID is needed alongside improved HR interventions to decrease HIV incidence. These interventions need a strong focus on decreasing sexual risk behaviours, with additional focus on individuals before they start injecting drugs because data suggest that new injectors already have a high prevalence of HIV. This should focus on interventions for PWUD as data suggest that most (81%) PWID smoked heroin before they started injecting [31] and PWUD can have a high prevalence of HIV (12%)[31]. Similarly, improved HR interventions are needed to address the HCV epidemic, with HCV treatment also being important for achieving HCV elimination. However, with HCV treatment not usually covered by health insurers, this is unlikely to happen in the near future [79], unless funded by international donors, such as the Global Fund in Kenya [80]. The results presented here show the benefits of scaling‐up existing interventions; further work is now needed to demonstrate their potential cost and cost‐effectiveness to show their potential value for money and to aid their prioritization. Interventions also need to be developed to reduce the sexual risk of PWID and PWUD, with most studies considering this being from high‐income countries [81].

## COMPETING INTERESTS

HF has received an honorarium from MSD unrelated to this research. JS reports non‐financial support from Gilead. PV and JGW have received unrestricted research funding from Gilead unrelated to this work. All other authors have no disclosures.

## AUTHORS' CONTRIBUTIONS

PV undertook the initial conceptualization with NL and BM, which was refined with EW. VS, PM, IJD, MAS, NoM, WK, MN and AR also contributed to the study aims. JS developed the initial model with input from JGW, which was adapted/extended for Tanzania by HF. HF performed model analyses and reviewed literature for any additional data. JS performed additional model analyses following initial submission. PV supervised the project. PM, BM, NoM and IJD provided oversight for the project in Tanzania. VS, PM, MAS, MN and HF provided data and/or undertook data analyses for the model. HF and PV wrote the first draft of the manuscript. All authors contributed to data interpretation, writing the manuscript, read and approved the final version.

## Supporting information


**Table S1**: Injecting risk behaviours among male and female injectors.
**Table S2**: Differences in reported sexual risk behaviours among male and female injectors.
**Table S3**: Risk behaviours among PWID and PWUD in the MdM survey. p‐values from univariable logistic regression.
**Figure S1**: HIV prevalence among (a) males and (b) females by duration of risk behaviour among all individuals in the MdM survey. The figure shows duration of sexual risk (black), duration of injecting risk (red) and overall duration of risk (blue), with data from the survey shown by dots and the linear regression line shown for each.
**Figure S2**: Box plots showing the median (centre line), interquartile range (box limits) and 2.5–97.5th percentiles (whisker limits) for (a) HIV prevalence among male PWID in 2019, (b) HIV prevalence among female PWID in 2019, (c) HCV prevalence among all PWID in 2019, (d) the percentage of sexual transmission among males, (e) the percentage of sexual transmission among females, (f) the percentage of HIV infections averted by 2019 by ART and harm reduction to date and (g) the percentage of HCV infections averted by 2019 by harm reduction to date. Boxes going left to right show this as the parameter sets are combined with the far right showing the median, IQR and 2.5–97.5th percentiles for the final parameter set used in all analysis.
**Figure S3**: The relative decrease in HIV incidence over time from 2019. For each year on the axis, the relative reduction in incidence from 2019 to that year was calculated. The figure shows for male PWID (blue line) and female PWID (orange line), respectively.
**Figure S4**: Box plot showing the relative change in HIV incidence among people who inject drugs (PWID) between 2019 and 2030 under different intervention scenarios. Note that boxes represent the median and 25th–75th percentile range and whiskers represent 2.5th–97.5th percentiles. Status quo is shown in blue shading while intervention scenarios are (orange shading) full harm reduction (Full HR; scaling up opioid agonist therapy (OAT) to 50% coverage and needle and syringe programmes (NSP) to 75% coverage), ART to high coverage and decrease sexual risk by 25%; (yellow shading) Full HR, ART to high coverage and decrease sexual risk by 30%; (purple shading) Full HR, ART to high coverage and decrease sexual risk by 75%.
**Figure S5**: Box plot showing the relative reduction in HIV incidence among people who inject drugs (PWID) between 2019 and 2030 under different intervention scenarios. Note that boxes represent the median and 25th–75th percentile range and whiskers represent 2.5th and 97.5th percentiles. Interventions scenarios by colour, with blue shading: scaling up opioid agonist therapy (OAT) to 50% coverage and needle and syringe programmes (NSP) to 75% coverage, denoted as full harm reduction (**Full HR**); orange shading: scaling‐up antiretroviral therapy (ART) to 81% coverage with 90% of those on ART virally suppressed; yellow shading: Full HR and ART to high coverage; purple shading: Full HR, ART to high coverage and decrease sexual risk by 75%.
**Figure S6**: Box plot showing the relative change in HCV incidence among people who inject drugs (PWID) between 2019 and 2030 under different intervention scenarios. Note that boxes represent the median and 25th–75th percentile range and whiskers represent 2.5th to 97.5th percentiles. Status quo is shown in blue shading while intervention scenarios are (orange shading) treat 5% of infected PWID with DAAs annually; ART to high coverage and decrease sexual risk by 25%; (yellow shading) treat 10% of infected PWID with DAAs annually; (purple shading) treat 15% of infected PWID with DAAs annually; (green sharing) full harm reduction (Full HR; scaling up opioid agonist therapy (OAT) to 50% coverage and needle and syringe programmes (NSP) to 75% coverage) and treating 5% of infected PWID with DAAs annually; (pale blue shading) Full HR and treating 10% of infected PWID with DAAs annually; (dark red shading) Full HR and treating 15% of infected PWID with DAAs annually.
**Figure S7**: Model projections for the (a) HIV prevalence amongst all PWID over time and (b) HCV prevalence amongst all PWID over time. The black line gives the median projections from 3500 parameter sets, with 95% credibility intervals shown in red shading. The grey dot‐dashed line shows the scenario where the rate of recruitment onto antiretroviral therapy is constant after 2011. Antiretroviral therapy started in 2004, OAT started in February 2011 and NSP started in March 2011. Grey circles and lines show the mean and 95% confidence interval of the data that the model was calibrated to, as given in Table S1. (c), the blue line shows the range used for model calibration.
**Figure S8**: Box plot showing the relative change in HIV and HCV incidence among people who inject drugs (PWID) between 2019 and 2030 under different intervention scenarios. Note that boxes represent the median and 25th–75th percentile range and whiskers represent 2.5th–97.5th percentiles. Status quo is shown in blue shading while intervention scenarios are (orange shading) scaling up opioid agonist therapy (OAT) to 50% coverage and needle and syringe programmes (NSP) to 75% coverage, denoted as full harm reduction (Full HR); (yellow shading) scaling‐up OAT to 50% coverage; (purple shading) scaling‐up NSP to 75% coverage.
ART refers to anti‐retroviral therapy; OAT stands for opioid agonist therapy; NSP stands for needle and syringe programmes; HCV stands for hepatitis C virus; PWID stands for people who inject drugs. HR stands for harm reduction; DAA stands for direct acting antivirals.
**Figure S9**: Model projections for (a) HIV prevalence and (b) HIV incidence. The black line gives the median projections for the status quo scenario, with 95% credibility intervals shown in grey shading. Median projections for intervention scenarios are as follows: (red) scaling up opioid agonist therapy (OAT) to 50% coverage and needle and syringe programmes (NSP) to 75% coverage, denoted as full harm reduction (Full HR); (blue) scaling‐up antiretroviral therapy (ART) to 81% coverage with 90% of those on ART virally suppressed; (green) Full HR and ART to high coverage; (purple) Full HR, ART to high coverage and decrease sexual risk by 50%.
**Figure S10**: Model projections for: (a) chronic HCV prevalence and (b) HCV incidence. The black line gives the median projections for the status quo scenario, with 95% credibility intervals shown in grey shading. Median projections for intervention scenarios are as follows: (red) scaling up opioid agonist therapy (OAT) to 50% coverage and needle and syringe programmes (NSP) to 75% coverage, denoted as full harm reduction (Full HR); (blue) treat 10% of HCV‐infected PWID with direct‐acting antivirals (DAAs) annually; (green) Full HR and treat 10% of HCV‐infected PWID with DAAs annually.
**Figure S11**: Box plot showing the relative decrease in HCV incidence among people who inject drugs (PWID) between 2019 and 2030 under different intervention scenarios. Note that boxes represent the median and 25th–75th percentile range and whiskers represent 2.5th–97.5th percentiles. Intervention scenarios are as follows: (blue shading) scale‐up OAT to 50% coverage and needle and syringe programmes (NSP) to 75% coverage , denoted as full harm reduction (Full HR); (red) treat 10% of HCV‐infected PWID with direct‐acting antivirals (DAAs) annually; (yellow) Full HR and treat 10% of HCV‐infected PWID with DAAs annually. Boxes in the left‐hand panel show results for all model runs; boxes in the middle panel show results for model runs which have decreasing HCV incidence in 2019; boxes in the right‐hand panel show results for model runs which have increasing HCV incidence in 2019.
**Figure S12**: Results of sensitivity analysis assuming 25% assortative mixing by harm reduction status. Model projections for the HIV prevalence amongst (a) male PWID and (b) female PWID over time; (c) HCV antibody prevalence amongst all PWID. For each, the black line gives the median projections from 3500 parameter sets, with 95% credibility intervals shown in red shading. Grey circles and lines show the mean and 95% confidence interval of the data that the model was calibrated to, as given in Table S1. In (c), the blue line shows the range used for model calibration.
**Figure S13**: Results of sensitivity analysis assuming 50% assortative mixing by harm reduction status. Model projections for the HIV prevalence amongst (a) male PWID and (b) female PWID over time; (c) HCV antibody prevalence amongst all PWID. For each, the black line gives the median projections from 3500 parameter sets, with 95% credibility intervals shown in red shading. Grey circles and lines show the mean and 95% confidence interval of the data that the model was calibrated to, as given in Table S1. In (c), the blue line shows the range used for model calibration.
**Table S4**: Relative change in HIV incidence among people who inject drugs (PWID) between 2019 and 2030 under different intervention scenarios for the baseline model fits, and sensitivity analyses which assume 25% or 50% assortative (like‐with like) mixing by harm reduction status. Cells show the median value with 95% CrI presented in parentheses.
**Table S5**: Relative change in HCV incidence among people who inject drugs (PWID) between 2019 and 2030 under different intervention scenarios for the baseline model fits, and sensitivity analyses which assume 25% or 50% assortative (like‐with like) mixing by harm reduction status. Cells show the median value with 95% CI presented in parentheses.
