# Supplementary material for: Modelling the impact of HIV and HCV prevention and treatment interventions for people who inject drugs in Dar es Salaam, Tanzania
Source: J Int AIDS Soc. 2021 Oct 18;24(10):e25817. doi: 10.1002/jia2.25817 (PMC8522890; doi:10.1002/jia2.25817)
Supplement: Supplementary file 1 — Table S1: Injecting risk behaviours among male and female injectors. Table S2: Differences in reported sexual risk behaviours among male and female injectors. Table S3: Risk behaviours among PWID and PWUD in the MdM survey. p‐values from univariable logistic regression. Figure S1: HIV prevalence among (a) males and (b) females by duration of risk behaviour among all individuals in the MdM survey. The figure shows duration of sexual risk (black), duration of injecting risk (red) and overall duration of risk (blue), with data from the survey shown by dots and the linear regression line shown for each. Figure S2: Box plots showing the median (centre line), interquartile range (box limits) and 2.5–97.5th percentiles (whisker limits) for (a) HIV prevalence among male PWID in 2019, (b) HIV prevalence among female PWID in 2019, (c) HCV prevalence among all PWID in 2019, (d) the percentage of sexual transmission among males, (e) the percentage of sexual transmission among females, (f) the percentage of HIV infections averted by 2019 by ART and harm reduction to date and (g) the percentage of HCV infections averted by 2019 by harm reduction to date. Boxes going left to right show this as the parameter sets are combined with the far right showing the median, IQR and 2.5–97.5th percentiles for the final parameter set used in all analysis. Figure S3: The relative decrease in HIV incidence over time from 2019. For each year on the axis, the relative reduction in incidence from 2019 to that year was calculated. The figure shows for male PWID (blue line) and female PWID (orange line), respectively. Figure S4: Box plot showing the relative change in HIV incidence among people who inject drugs (PWID) between 2019 and 2030 under different intervention scenarios. Note that boxes represent the median and 25th–75th percentile range and whiskers represent 2.5th–97.5th percentiles. Status quo is shown in blue shading while intervention scenarios are (orange shading) full harm reduction (F [file JIA2-24-e25817-s001.docx]

# Supplementary Materials for: Modelling the impact of prevention and treatment interventions for people who inject drugs in Dar es Salaam, Tanzania

Authors: Hannah Fraser^1*^, Jack Stone^1^, Ernst Wisse^2^, Veryeh Sambu^3^, Peter Mfisi^4^, Ivan J Duran^2^, Mireia Aguirre Soriano^2^, Josephine G. Walker^1^, Nobelrich Makere^5^, Niklas Luhmann^2^, William Kafura^6^, Maieule Nouvellet^2^, Allan Ragi^7^, Ben Mundia^7^, Peter Vickerman^1^

1. Population Health Sciences, Bristol Medical School, University of Bristol, U
2. Médecins du Monde, France
3. National AIDS Control Programmes, Dar es Salaam, Tanzania
4. The Drug Control and Enforcement Authority, Prime Ministers Office, Tanzania
5. Tanzania Council for Social Development (TACOSODE), Dar es Salaam, Tanzania
6. Tanzania Commission for AIDS (TACAIDS), Dar es Salaam, Tanzania
7. Kenya AIDS NGO Consortium, Kenya

* Corresponding Author

Email: [hannah.fraser@bristol.ac.uk](mailto:hannah.fraser@bristol.ac.uk)

# Methods

## Model description

We developed a dynamic, deterministic compartmental HIV and HCV transmission model amongst PWID to evaluate the impact of existing and scaled-up interventions in Dar es Salaam, Tanzania. The model incorporates HIV and HCV transmission due to injecting drug use (IDU) alongside HIV transmission due to sexual risk behaviour. The population is stratified by gender, HIV infection state (susceptible; acute infection; chronic infection; pre-AIDS; AIDS (Main paper – Figure 1a)), HIV treatment status (on/off ART (Main paper – Figure 1a)), HCV infection state (susceptible; previously exposed (Ab+, RNA-); chronic infection (Ab+, RNA+); on HCV treatment (Main paper – Figure 1b)) and harm reduction state (off OAT and NSP, OAT or NSP only, or on both, (Main paper – Figure 1c)).

Individuals enter the model through initiation of injecting drug use, susceptible to HCV, and not accessing OAT or NSP. A proportion of those entering the model are chronically infected with HIV, while the remainder enter as susceptible to HIV. Entry into the model is set to balance individuals leaving the model due to cessation of injecting drug use and non-HIV related death, resulting in a decreasing PWID population size due to HIV-related death. Male and female PWID leave the model in all compartments through cessation of injecting and non-HIV or HCV related death. HIV related mortality is accounted for through additional mortality in the AIDS compartment. We assume no additional mortality due to HCV-related death due to all PWID initiating injecting as susceptible, the short duration of injecting (3 – 14years) and the long time-frame for HCV disease progression (around 20 years).

Susceptible PWID become infected with HIV and HCV through sharing of injecting equipment amongst PWID (injecting transmission), with HIV also being sexually transmitted through contacts with other PWID or non-PWID (sexual transmission). Injecting behaviour data and patterns of HCV suggest that male and female PWID have similar injecting risks (Supplementary Table 1), and therefore we assume that HIV and HCV transmission through injecting does not vary by gender. For sexual HIV transmission, we only model heterosexual HIV transmission as data suggests few male PWID have sex with men (5.6% (3.5-8.5%) in the past 12 months[1]). Due to broad differences in the prevalence of HIV between male and female PWID (main paper Table 1) and differences in reported sexual risk behaviours, we assume that the risk of sexual HIV transmission differs by gender (Supplementary Table 2). We assume that a PWID’s sexual partners can be with other PWID or the general population, with the model assuming the same sexual risk by whether their sexual partner is a PWID or not.

***Supplementary Table 1:*** *Table showing injecting risk behaviours among male and female injectors*

|  | Males | Females | Reference |
| --- | --- | --- | --- |
| Average age of injectors | 30 (IQR: 26-35) | 29 (IQR: 25-32) | [1] |
| Average number of times injected in the past 30 days | 100.8 | 92.4 | [2] |
| Mean times injected, last 30 days | 29.3 | 29.9 | [3] |
| Percentage of times used a new needle at last injecting (Y/N)* | 96.4 (SD: 10.39) | 95.5 (SD: 13.67) | [2] |
| Percentage that at the last injection used a syringe/needle that was previously used by someone else (not cleaned)* | 5% | 6% | [1] |

*these percentages would add to one if from the same dataset, but they are from different datasets

***Supplementary Table 2:*** *Table showing differences in reported sexual risk behaviours among male and female injectors.*

|  | Males | Females | Reference |
| --- | --- | --- | --- |
| Had sex in the last 30 days | 58.2%  (51.7 – 64.6%) | 93.4%  (87.6 – 97.2%) | [3] |
| Proportion of PWID’s partners that are PWID | 0.165  (0.103 – 0.246) | 0.467  (0.253 – 0.657) | [1] |
| Traded sex for money | < 1% | 83.7%  (76.0 – 89.8%) | [3] |
| Used a condom at last sex | 19.6%  (15.6 – 24.1%) | 35.6%  (24.7 – 47.7%) | [1] |
| Casual sexual partner(s) in previous 12 months | 52.0%  (45.9 – 58.0%) | 28.2%  (18.1 – 40.1%) | [1] |

Susceptible PWID can become infected with HIV through sexual contacts with other PWID or the general population. In each case the force of infection is dependent upon the time-varying HIV prevalence amongst PWID or the general population males and females based on data from UNAIDS, the time varying ART coverage amongst PWID or the general population and the effectiveness of ART at reducing HIV sexual transmission. Susceptible PWID also become infected with HIV through injecting transmission; the force of infection is dependent on the HIV transmission rate, proportion of male and female PWID in each HIV infection stage and the infectivity of each stage of infection[4]. The baseline injecting transmission rate for PWID not accessing harm reduction interventions is decreased by fixed multiplicative cofactors for PWID on OAT, NSP or both based on recent systematic reviews[5, 6]. PWID mix randomly to form potential transmission contacts with other PWID.

Following HIV infection, individuals enter a short acute phase of infection before progressing to the chronic infection phase at a constant rate. Individuals then progress to Pre-AIDS and AIDS phases at fixed rates. We assume that individuals in the acute and pre-AIDS phases of infection are more infectious than those chronically infected[4]. Individuals in the AIDS phase are assumed only to engage in injecting and sexual behaviour if they are on ART and are as infectious as those in the pre-AIDS stage of infection on ART. Individuals leave the AIDS phase due to HIV-related mortality. Individuals in chronic, pre-AIDS or AIDS phases of infection can be enrolled onto ART at a time-varying rate, whereupon infectiousness is reduced compared to chronically infected individuals not receiving ART depending upon levels of viral suppression and average viral load, based on a study in Uganda which found that HIV transmission was rare among people with viral load levels of less than 1,500 copies of HIV-1 RNA per milliliter[7]. Individuals enrolled on ART progress through disease stages at a slower rate than those not on ART based on survival rates of individuals on ART, as is the HIV-related mortality rate. We assume that PWID receiving ART can be lost to follow-up and then re-enrolled onto ART at the same rate as ART-naïve infected PWID.

PWID can become infected with HCV through sharing of injecting equipment. We do not model sexual transmission of HCV as we assume the primary route of HCV transmission among PWID is the sharing of equipment for the preparation and injection of drugs. HCV transmission is also dynamic and simulated at a per-capita transmission rate dependent on the current prevalence of chronic HCV infection in PWID. As for HIV, the baseline HCV transmission rate for PWID not on OAT or NSP is decreased by fixed multiplicative cofactors for PWID on OAT, NSP or both[8]. We assume that PWID co-infected with HIV and HCV are more infectious than those with HCV mono-infection[9].

Following infection, a proportion of PWID spontaneously clear infection and enter the previously exposed compartment (antibody (Ab) + and RNA-), with the remainder progressing to chronic infection (Ab+ and RNA+). The proportion spontaneously clearing infection differs according to HIV status[10, 11]. Although there is currently no HCV treatment among PWID in Dar es Salaam, we assume for the scale-up scenario that a proportion of chronically infected PWID can be treated annually, with treatment having an average duration of 12 weeks[12]. A proportion of treated individuals are assumed to achieve a sustained viral response (SVR, effective cure) and enter the previously exposed compartment. Those who do not achieve SVR return to the chronically infected HCV compartment where they can be re-treated. We assume re-treatment occurs at the same rate as for treatment naïve individuals.

PWID are enrolled on OAT and NSP at time-varying rates and leave OAT and NSP at constant rates. For the baseline status quo scenario, we assume PWID can only access OAT or NSP, but not both, although this is changed in the scale up scenarios. It is assumed that enrolment and leaving rates for OAT and NSP are independent of HIV and HCV status. However, those on OAT have an increased rate of starting and reduced loss-to-follow-up of ART, and an increased likelihood of being virally supressed if also on ART[13].

## Model parameterisation and calibration

The model was calibrated to detailed data for Dar es Salaam. This included data from a Médecins du Monde (MdM) harm reduction programme, which started in late 2010, and includes NSP provision[1]. This comprised detailed information on the number of PWID accessing NSP at fixed sites and within the community on a monthly basis from January 2016 to August 2018, and results from a bio-behavioural survey from June 2010 which included snowball and targeted sampling (n=267). Data was also obtained from Integrated Bio-Behavioural Surveillance (IBBS) surveys undertaken in 2014 and 2017, which employed respondent driving sampling[14]. Routinely collected data from OAT clinics within Dar es Salaam was also used[15-18]. Government data on the cumulative number of PWID accessing OAT at clinics in Dar es Salaam, and data on the proportion of PWID on ART and viral suppression data were also used.

We assume that injecting drug use started between 1998 and 2001[6], and that the PWID population size in Dar es Salaam is currently between 9,000-15,000[19]. ART is assumed to start in 2004 and scales up until 2011 whereupon it remains constant. We determined the rate of LTFU amongst PWID on ART based on 53.5% retention on ART after 36 months in the general population[20] and assuming an increased relative risk (1.36; 95%CI: 1.22–1.52) of LTFU among PWID compared to the general population based on a 34 multi-country study[21]. The first OAT clinic started in Dar es Salaam in February 2011 and we assume that 2,099 PWID had been enrolled up to 2014, with 651, 968, 1,100 and 436 enrolled in the years 2015, 2016, 2017 and 2018 leading to a total of 5,254 PWID ever enrolled on OAT by August 2018 and ~3,300 currently on OAT (Government data for this project). NSP was initiated in March 2011, and we assume coverage increased until April 2017 whereupon it remains constant. The total number of PWID reached each month through the community and fixed sites ranged from 861 to 1,692 over2016-2018, however data from March-August 2018 suggests that 12% of PWID access both the community and fixed site in a given month. Therefore, we assume that the NSP reaches between 753 and 1,479 individuals each month.

Based on the MdM Bio-Behavioural survey we assume that males and females have been sexually active for between 7.5yrs (6.7-8.3yrs) and 7.9yrs (6.0-9.9yrs) prior to injecting drugs, respectively, and use this to calculate (a) the possible rate of sexual transmission that would be required to achieve the prevalence amongst new injectors and (b) the additional injecting transmission required to achieve the overall prevalence among male and female injectors.

To determine the prevalence amongst new injectors, we used data from the MdM survey which recruited people who use drugs (PWUD) as well as PWID. Data analysed showed similar sexual risk behaviours between PWUD and PWID (Supplementary Table 2) – therefore we estimated HIV prevalence among PWID when they start injecting using data from PWID and PWUD from the MdM dataset. We considered HIV prevalence by duration of (a) sexual risk, (b) injecting risk (assumed to be 0 for PWUD) and (c) overall risk (found by calculating the duration of the longest risk behaviour of each individual). In males, HIV prevalence by duration of sexual risk and by duration of overall risk are very similar (hence many start sexual activity before injecting activity), with there also being a similar HIV prevalence for those with a low duration of injecting risk and prevalence increasing as duration of injecting risk increases (Supplementary Figure 1a). While the trends are not as clear among females (Supplementary Figure 1b), trends still show greater prevalence by duration of injecting risk than for sexual and overall risk. Therefore, we performed a logistic regression on HIV prevalence by duration of sexual risk, duration of injecting risk, and gender, and use the output to determine the prevalence of new injectors in the model. Using the average duration of sexual risk before initiating injecting given above, we are able to estimate HIV prevalence among new male and female injectors as 0.3–5.3% and 1.3–45.4%, respectively.

***Supplementary Table 3****: Risk behaviours among PWID and PWUD in the MdM survey. P-values from univariable logistic regression.*

|  | PWID | | | | PWUD | | | |  |
| --- | --- | --- | --- | --- | --- | --- | --- | --- | --- |
|  | n | N | Mean | 95%CI | n | N | Mean | 95%CI | p-value (χ^2^ test) |
| Sexual intercourse in past 12 months | 202 | 265 | 76.2% | 70.6 - 81.2% | 141 | 160 | 88.1% | 82.1 - 92.7% | 0.003 |
| Among those having sex in the past 12 months, sexual intercourse in the past month | 106 | 201 | 52.7% | 45.6 - 59.8% | 86 | 141 | 61.0% | 52.4 - 69.1% | 0.130 |
| Among those having sex in the past 12 months, have had sex with spouse/live-in partner | 144 | 201 | 71.6% | 64.9 - 77.8% | 102 | 141 | 72.3% | 64.2 - 79.5% | 0.887 |
| Condom use among those having sex with spouse/live-in partner | 20 | 143 | 14.0% | 8.8 - 20.8% | 14 | 101 | 13.9% | 7.8 - 22.2% | 0.978 |
| Among those having sex in the past 12 months, have had commercial sex | 79 | 196 | 40.3% | 33.4 - 47.5% | 66 | 140 | 47.1% | 38.7 - 55.8% | 0.212 |
| Condom use among those having commercial sex | 35 | 73 | 48.0% | 36.1 - 60.0% | 30 | 65 | 46.2% | 33.7 - 59.0% | 0.833 |
| Among those having sex in the past 12 months, casual partners | 98 | 202 | 48.5% | 41.4 - 55.6% | 63 | 141 | 44.7% | 36.3 - 53.3% | 0.484 |
| Condom use with casual partners | 28 | 87 | 32.2% | 22.6 - 43.1% | 15 | 59 | 25.4% | 15.0 - 38.4% | 0.379 |
| Used a condom for last sex act among those having sex in the past 12 months | 45 | 199 | 22.6% | 17.0 - 29.1% | 31 | 141 | 22.0% | 15.5 - 29.7% | 0.891 |
| Among those who have had commercial partnerships - sold sex | 39 | 78 | 50.0% | 38.4 - 61.5% | 37 | 65 | 56.9% | 44.0 - 69.2% | 0.409 |
| Among those who have had commercial partnerships - bought sex | 61 | 79 | 77.2% | 66.4 - 85.9% | 43 | 65 | 66.2% | 53.4 - 77.4% | 0.140 |
| MEN ONLY |  |  |  |  |  |  |  |  |  |
| Ever had any male partners | 13 | 231 | 5.63% | 3.0 - 9.4% | 7 | 125 | 5.60% | 2.3 - 11.2% | 0.991 |
| Out of those ever having sex with men, have had anal intercourse in past 12 months | 11 | 11 | 100% | 71.5 – 100% | 5 | 6 | 83.33% | 35.9 - 99.6% | 0.163 |

***Supplementary Figure 1****: HIV prevalence among (a) males and (b) females by duration of risk behaviour among all individuals in the MdM survey. The figure shows duration of sexual risk (black), duration of injecting risk (red) and overall duration of risk (blue), with data from the survey shown by dots and the linear regression line shown for each.*

(a) (b)


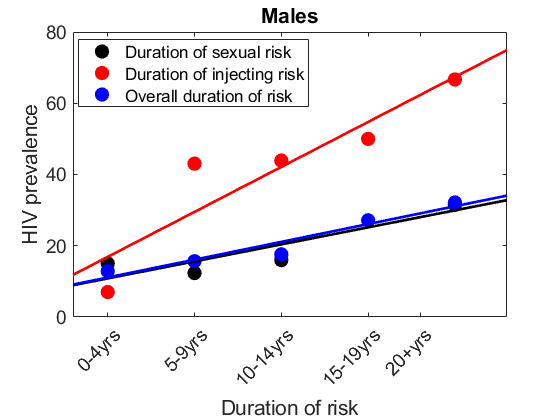

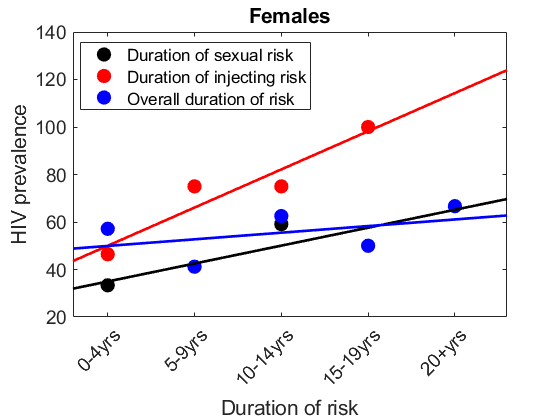


The model was calibrated using an approximate Bayesian computation Sequential Monte Carlo (ABC SMC) method[22] to data on the: (1) HIV prevalence amongst male and female PWID from the MdM survey[1], several other bio-behavioural studies undertaken in different districts of Dar es Salaam amongst PWID[23-25], and the IBBS surveys[14]; (2) HCV antibody prevalence amongst PWID from OAT clinics,[18] the MdM survey[1] and an IBBS survey in 2017[14]; (3) self-reported ART coverage amongst HIV positive PWID from the MdM survey and PWID attending OAT clinics[1, 18, 26]; (4) programme data on the cumulative number of PWID enrolled onto OAT; (5) programme data on the number of PWID recently in contact with NSP; (6) PWID population size estimates[19]; (7) survey data on the proportion of PWID that are female[15, 18] and (8) bounds for the sexual HIV incidence among male and female PWID. Table 1 (Main paper) shows the calibration data used in the SMC routine. During the ABC SMC, 5,000 parameter sets were sampled from prior probability distributions (Main paper – Table 2). These were then perturbed and resampled in an iterative process to achieve 5,000 parameter sets where

- The proportion of PWID that were female and the number of PWID on NSP were between the bounds (see Table 1)
- The mean square difference of the number of people on OAT between the model and data estimates were improved on by < 2% of the previous iteration.
- The likelihood of the model estimating HIV prevalence among males and females, and ART coverage were improved on by <2% of the previous iteration.
- HCV prevalence among all PWID was between the bounds (see Table 1)
- Sexual HIV incidence matched what would be expected based on prevalence when individuals start injecting.

We conducted the ABC SMC multiple times, independent of each other with different random seeds, each time producing a set of 5,000 accepted parameter sets according to the above criteria, until the median of key model projections converged (were <5% of the previous combined sets). These key model projections were:

1. HIV prevalence among male PWID in 2019
2. HIV prevalence among female PWID in 2019
3. HCV prevalence among PWID in 2019
4. % of new HIV infections among male PWID in 2019 that is due to sexual transmission
5. % of new HIV infections among female PWID in 2019 that is due to sexual transmission
6. % of HIV infections averted by ART and harm reduction to date
7. % of HCV infections averted by harm reduction to date

We then weighted each parameter set based on the likelihood of that parameter set for HIV prevalence and ART coverage, and sampled 10% of the weighted parameter sets. In this case we ran 7 iterations of the ABC SMC code, producing 35,000 parameters sets. Therefore, we had 3,500 final parameter sets. Supplementary Figure 2 shows how the results converged as each parameter set has been added, as well as the results for the final parameter set.

***Supplementary Figure 2:*** *Box plots showing the median (centre line), interquartile range (box limits) and 2.5-97.5^th^ percentiles (whisker limits) for (a) HIV prevalence among male PWID in 2019, (b) HIV prevalence among female PWID in 2019, (c) HCV prevalence among all PWID in 2019, (d) the percentage of sexual transmission among males, (e) the percentage of sexual transmission among females, (f) the percentage of HIV infections averted by 2019 by ART and harm reduction to date and (g) the percentage of HCV infections averted by 2019 by harm reduction to date. Boxes going left to right show this as the parameter sets are combined with the far right showing the median, IQR and 2.5-97.5^th^ percentiles for the final parameter set used in all analysis.*

# Model equations

Let $X_{i,j,k}^{m,n}$ be the number of PWID in the model where

- subscript $i$ denotes gender ($i=1$, male; $i=2$, female)
- subscript $j$ denotes harm reduction intervention status ($j=1$, not accessing OAT or NSP, never been on OAT; $j=2$, accessing OAT only; $j=3$, accessing NSP only, never been on OAT; $j=4$, accessing both OAT and NSP; $j=5$, not accessing OAT or NSP, ever been on OAT; $j=6$, accessing NSP only, ever been on OAT)
- subscript $k$ denotes ART treatment ($k=1$, not on ART; $k=2$, on ART)
- superscript $m$ denotes HIV infection status ($m=1$, susceptible to HIV; $m=2$, acute HIV infection; $m=3$, chronic HIV infection, $m=4,$ pre-AIDS phase of infection; $m=5$, AIDS phase of infection
- superscript $n$ denotes HCV infection status ($n=1$, susceptible to HCV; $n=2$, exposed/previously infected with HCV (Ab+, RNA -ve); $n=3$, chronic HCV infection (Ab+, RNA +ve); $n=4$, HCV treatment)

The ordinary differential equation models can be written as

$$\frac{dX_{i,j,k}^{m,n}}{dt}=\Theta_{i,j,k}^{m,n}+\Sigma_{i,j,k}^{m,n}+\Lambda_{i,j,k}^{m,n}+P_{i,j,k}^{m,n}+K_{i,j,k}^{m,n}+{\Pi_{i,j,k}^{m,n}+M}_{i,j,k}^{m,n}$$

where

- $\Theta_{i,j,k}^{m,n}$represents recruitment of PWID into the model
- $\Sigma_{i,j,k}^{m,n}$ represents transitions between harm reduction states (OAT and NSP)
- $\Lambda_{i,j,k}^{m,n}$ represents HIV transmission
- $P_{i,j,k}^{m,n}$ represents HIV disease progression
- $K_{i,j,k}^{m,n}$ represents transitions on and off ART
- $\Pi_{i,j,k}^{m,n}$ represents HCV transmission and treatment
- $M_{i,j,k}^{m,n}$ represents non-HIV related mortality from the model and injecting cessation

Each of these terms are described in more detail below.

### Inflow of injectors - $\Theta_{i,j,k}^{m,n}$

The inflow of new injectors is given by

$$\Theta_{i,1,1}^{1,1}=\left( 1-p_{i} \right)\theta_{i}\left( t \right)$$

$$\Theta_{i,1,1}^{3,1}=p_{i}\left( 1-q \right)\theta_{i}(t)$$

$$\theta_{i,1,2}^{3,1}=p_{i}q\theta_{i}(t)$$

where

- $p_{i}$ is the HIV prevalence among PWID of gender $i$ when they initiate injecting
- $\theta_{i}$ is the number of new PWID of gender $i$ who enter the model at time $t$ – note this is set to equal the number of leave the model due to non-HIV related mortality.
- $q$ is the proportion of HIV positive new PWID who are on ART.

Note that for all other combinations of $i,j,k,m,n$ not listed above $\Theta_{i,j,k}^{m,n}=0$.

### Transitions between harm reduction states - $\Sigma_{i,j,k}^{m,n}$

Transitions between harm reduction states are given by:

$$\Sigma_{i,1,k}^{m,n}=-\left( \kappa_{a}+\beta_{a} \right)X_{i,1,k}^{m,n}+\epsilon X_{i,3,k}^{m,n}$$

$$\Sigma_{i,2,k}^{m,n}=-\left( R_{i}\gamma+\beta_{b} \right)X_{i,2,k}^{m,n}+\kappa_{a}(X_{i,1,k}^{m,n}+X_{i,5,k}^{m,n})+\epsilon X_{i,4,k}^{m,n}$$

$$\Sigma_{i,3,k}^{m,n}=-\left( \epsilon+\kappa_{b} \right)X_{i,3,k}^{m,n}+\beta_{a}X_{i,1,k}^{m,n}$$

$$\Sigma_{i,4,k}^{m,n}=-\left( \epsilon+R_{i}\gamma\right)X_{i,4,k}^{m,n}+\kappa_{b}(X_{i,3,k}^{m,n}+X_{i,6,k}^{m,n})+\beta_{b}X_{i,2,k}^{m,n}$$

$$\Sigma_{i,5,k}^{m,n}=-\left( \kappa_{a}+\beta_{a} \right)X_{i,5,k}^{m,n}+R_{i}\gamma X_{i,2,k}^{m,n}+\epsilon X_{i,6,k}^{m,n}$$

$$\Sigma_{i,6,k}^{m,n}=-\left( \epsilon+\kappa_{b} \right)X_{i,6,k}^{m,n}+R_{i}\gamma X_{i,4,k}^{m,n}+\beta_{a}X_{i,5,k}^{m,n}$$

where

- $\kappa_{a}$ and $\kappa_{b}$ denote the rate of recruitment onto OAT. Note that for all baseline runs $\kappa_{b}=0$ as individuals cannot access both OAT and NSP.
- $\beta_{a}$ and $\beta_{b}$ denote the rate of recruitment onto NSP. Note that for all baseline runs $\beta_{b}=0$ as individuals cannot access both OAT and NSP.
- $\gamma$ denotes the OAT leaving rate.
- $\epsilon$ denotes the NSP leaving rate.
- $R$ denotes the adjusted relative risk of increased loss to follow-up in females compared to males (i.e. $R_{1}=1$ and $R_{2}$ can be found in Table 2 in the main paper).

### HIV transmission - $\Lambda_{i,j,k}^{m,n}$

The terms in this expression are concerned with HIV transmission and are given by

$$\Lambda_{i,j,1}^{1,n}=-\left( \Lambda_{i,j}^{sex}+\Lambda_{j}^{inj} \right)X_{i,j,1}^{1,n}$$

$$\Lambda_{i,j,1}^{2,n}=\left( \Lambda_{i,j}^{sex}+\Lambda_{j}^{inj} \right)X_{i,j,1}^{1,n}$$

$\Lambda_{i,j,k}^{m,n}=0$ if $m\geq3$; $\Lambda_{i,j,2}^{1,n}=0; \Lambda_{i,j,2}^{2,n}=0$

where

- $\Lambda_{i,j}^{sex}$ is the HIV sexual force of infection for PWID of gender $i$ in harm reduction state $j$ (see section below).
- $\Lambda_{j}^{inj}$ is the HIV injecting force of infection for PWID in harm reduction state $j$ (see section below).

**HIV injecting force of infection**

The HIV injecting force of infection for PWID in each intervention state $j$ is denoted by $\Lambda_{j}^{inj}$ and is given by

$$\Lambda_{j}^{inj}={\phi_{j}^{HIV}\beta}_{inj}^{HIV}Y$$

where

$$Y=\frac{\sum_{i} \sum_{n} \sum_{j} Y_{j}}{\sum_{i} \sum_{n} \sum_{j} Z_{j}}$$

and

$$Y_{j}={\Phi_{j}^{HIV}[\phi_{A}X}_{i,j,1}^{2,n}+X_{i,j,1}^{3,n}+\phi_{P}X_{i,j,1}^{4,n}+\delta_{j}^{inj}(X_{i,j,2}^{3,n}+\phi_{P}{(X}_{i,j,2}^{4,n}+X_{i,j,2}^{5,n}))]$$

$$Z_{j}=\Phi_{j}^{HIV}\left[ \sum_{m=1:4} X_{i,j,1}^{m,n}+\sum_{m=2:5} X_{i,j,2}^{m,n} \right]$$

where

- $\beta_{inj}^{HIV}$ denotes the HIV transmission rate for injecting for PWID who are not accessing OAT or NSP and are not currently on ART.
- $\Phi_{j}^{HIV}$ denotes the relative reduction in HIV transmission for injecting transmission if accessing OAT ($j=2$), NSP $(j=3,6)$ or both $\left( j=4 \right)$. Note that when not accessing OAT or NSP $(j=1,5)$ $\Phi_{j}^{HIV}=1$.
- $\phi_{A}$ denotes the relative increase in HIV transmissibility if in the acute stage of infection compared to the latent stage of infection.
- $\phi_{P}$ denotes the relative increase in HIV transmissibility if in the pre-AIDS stage of infection (or AIDS stage when on ART) compared to the latent stage of infection.
- $\delta_{j}^{inj}$ denotes the average reduction in HIV transmission through injecting by ART for PWID off OAT $(j=1,3,5,6)$ or on OAT ($j=1,2$). Note that $\delta_{j}$ is explained further below.

**HIV sexual force of infection**

The HIV sexual force of infection for PWID of gender $i$ is denoted by $\Lambda_{i}^{sex}$ and is given by

$$\lambda_{1,j}^{sex}={\beta_{2}^{sex}(q}_{1}Q_{2}+\left( 1-q_{1} \right)r_{2}(1-g+g\delta_{1}^{sex})$$

$$\lambda_{2,j}^{sex}=\beta_{1}^{sex}(q_{2}Q_{1}+\left( 1-q_{2} \right)r_{1}(1-g+g\delta_{2}^{sex})$$

where

$$Q_{i}=\frac{\sum_{n} \sum_{j} L_{i,j}^{N}}{\sum_{n} \sum_{j} L_{i,j}^{D}}$$

and

$$L_{i,j}^{N}={\phi_{A}X}_{i,j,1}^{2,n}+X_{i,j,1}^{3,n}+\phi_{P}X_{i,j,1}^{4,n}+\delta_{j}^{inj}(X_{i,j,2}^{3,n}+\phi_{P}{(X}_{i,j,2}^{4,n}+X_{i,j,2}^{5,n}))$$

$$L_{i,j}^{D}=\sum_{m=1:4} X_{i,j,1}^{m,n}+\sum_{m=2:5} X_{i,j,2}^{m,n}$$

where

- $\beta_{i}^{sex}$ denotes the sexual HIV transmission rate from group $i$ to the opposite gender for PWID who are not currently on ART.
- $q_{i}$ is the proportion of sexual partners of group $i$ that are with other PWID.
- $r_{i}$ is the HIV prevalence among general population of gender $i$
- $g$ is the ART coverage in the general population
- $\delta_{j}^{sex}$ denotes the average reduction in HIV transmission through sexual risk by ART for PWID off OAT $(j=1,3,5,6)$ or on OAT ($j=2,4$). Note that $\delta_{j}$ is explained further below.

Effectiveness of ART for reducing HIV sexual and injecting transmission

If $V_{j}^{S}=p_{s} (j=1,3,5,6)$ denotes the proportion of PWID on ART but not OAT who are virally supressed, and $r_{s}$ denotes the odds ratio of viral suppression when on OAT compared to PWID not on OAT then the proportion of PWID on ART with viral suppression when on OAT is given by

${V_{j}^{S}=p}_{s}^{*}=\frac{p_{s}r_{s}}{1+p_{s}\left( r_{s}-1 \right)},$ $j=2,4$.

To determine the decrease in HIV transmission risk among virally suppressed and unsuppressed PWID on ART, we estimated the log difference between the baseline plasma viral load ($PVL-v_{b})$ for PWID off ART, and PWID on ART with suppressed ($v_{s})$ PVL $\Delta_{s}=v_{b}-v_{s}$, or unsuppressed ($v_{u})$ PVL$\Delta_{u}={v_{b}-v}_{u}$. Because prior studies [27, 28] suggest HIV transmission risk increases (factor $r_{t}$) for each log increase in PVL, these log differences in PVL were used to estimate the relative decrease in transmission risk among virally suppressed $(\delta^{S})$ and unsuppressed $(\delta^{N})$ PWID on ART:

$$\begin{aligned} \delta^{S}=1/{(r}_{t}^{\Delta_{s}}) \\ \delta^{N}=1/{(r}_{t}^{\Delta_{u}}) \end{aligned}$$

The average reduction in HIV sexual transmission by ART for PWID off OAT $(j=1,3,5,6)$ or on OAT $(j=2,4)$ is then given by

$${\delta_{j}^{sex}=V}_{j}^{S}\delta^{S}+\left( 1-V_{j}^{S} \right)\delta^{N}$$

If $F_{inj}$ is the relative effectiveness on viral suppression of ART for injecting compared to sexual transmission then the average reduction in HIV injecting transmission for PWID off $(j=1,3,5,6)$ or on OAT $(j=2,4)$ is then given by

$$\delta_{j}^{inj}=1-F_{inj}(1-(V_{j}^{S}\delta^{S}+\left( 1-V_{j}^{S} \right)\delta^{N})$$

### HIV disease progression - $P_{i,j,k}^{m,n}$

These terms are concerned with HIV disease progression and are denoted by $P_{i,j,k}^{m,n}$. Disease progression for those not on ART $(k=1)$ is given by

$$P_{i,j,k}^{2,n}=-\tau_{A}X_{i,j,k}^{2,n}$$

$$P_{i,j,k}^{3,n}=\tau_{A}X_{i,j,k}^{2,n}-{\tau_{E}^{k}\tau}_{C}X_{i,j,k}^{3,n}$$

$$P_{i,j,k}^{4,n}={\tau_{E}^{k}\tau}_{C}X_{i,j,k}^{3,n}-\tau_{E}^{k}\tau_{P}X_{i,j,k}^{4,n}$$

$$P_{i,j,k}^{5,n}={\tau_{E}^{k}\tau}_{P}X_{i,j,k}^{4,n}-\tau_{E}^{k}\tau_{I}X_{i,j,k}^{5,n}$$

where

- $\tau_{A}$ is the rate of progressing from acute HIV infection to latent HIV infection
- $\tau_{C}$ is the rate of progressing from latent HIV infection to pre-AIDS HIV infection if not on ART
- $\tau_{P}$ is the rate of progressing from pre-AIDS HIV infection to AIDS if not on ART
- $\tau_{I}$ is mortality due to AIDS if not on ART
- $\tau_{E}^{k}$ is the relative reduction in HIV disease progression and mortality due to AIDS if on ART (note that $\tau_{E}^{1}=1$).

### Transitions on and off ART - $K_{i,j,k}^{m,n}$

We assume that those in the latent, pre-AIDS and AIDS phases of HIV infection are able to enrol on ART and are lost-to follow-up from ART.

$$K_{i,j,1}^{m,n}=-O_{j}\eta X_{i,j,1}^{m,n}+L_{j}\zeta X_{i,j,2}^{m,n} if m=3,4,5$$

$$K_{i,j,2}^{m,n}=O_{j}\eta X_{i,j,1}^{m,n}-L_{j}\zeta X_{i,j,2}^{m,n} if m=3,4,5$$

$$K_{i,j,k}^{m,n}=0 if m=1,2$$

where

- $\eta$ is the rate of enrolment onto ART if in the latent HIV, pre-AIDS and AIDS stages of infection.
- $\zeta$ is the rate of loss to follow up from ART if in the latent HIV, pre-AIDS and AIDS stages of infection
- $O_{j}$ is the effect of being on OAT on ART enrolment (i.e. $O_{j}=1$ when $j=1,3,5,6$).
- $L_{j}$ is the effect of being on OAT on loss-to follow-up of ART ((i.e. $L_{j}=1$ when $j=1,3,5,6$).

### HCV transmission and treatment - $\Pi_{i,j,k}^{m,n}$

These terms are concerned with HCV transmission and are denoted by $\Pi_{i,j,k}^{m,n}$

$$\Pi_{i,j,k}^{m,1}=-\beta_{j}X_{i,j,k}^{m,1} \forall m$$

$$\Pi_{i,j,k}^{1,2}=-\left( 1-\alpha_{-} \right)\beta_{j}X_{i,j,k}^{1,2}+\alpha_{-}{\beta_{j}X}_{i,j,k}^{1,1}+s\omega X_{i,j,k}^{1,4}$$

$$\Pi_{i,j,k}^{m,2}=-\left( 1-\alpha_{+} \right)\beta_{j}X_{i,j,k}^{m,2}+\alpha_{+}\beta_{j}X_{i,j,k}^{m,1}+s\omega X_{i,j,k}^{m,4} m\geq2$$

$$\Pi_{i,j,k}^{1,3}=\left( 1-\alpha_{-} \right)\beta_{j}\left( X_{i,j,k}^{1,1}+X_{i,j,k}^{1,2} \right)-\Psi X_{i,j,k}^{1,3}+\left( 1-s \right)\omega X_{i,j,k}^{1,4}$$

$$\Pi_{i,j,k}^{m,3}=\left( 1-\alpha_{+} \right)\beta_{j}\left( X_{i,j,k}^{m,1}+X_{i,j,k}^{m,2} \right)-\Psi X_{i,j,k}^{m,3}+\left( 1-s \right)\omega X_{i,j,k}^{m,4} m\geq2$$

$$\Pi_{i,j,k}^{m,4}=\Psi X_{i,j,k}^{m,3}-\omega X_{i,j,k}^{m,4} \forall m$$

where

- $\beta_{j}$ is the HCV force of infection for individuals accessing harm reduction state $j$.
- $\alpha_{-}$ is the proportion of HCV infections that spontaneously clear among HIV negative PWID.
- $\alpha_{+}$ is the proportion of HCV infections that spontaneously clear among HIV positive PWID.
- $\Psi$ is the HCV treatment rate
- $s$ is the sustained viral response rate among those undertaking HCV treatment
- $1/\omega$ is the average duration of HCV treatment.

HCV force of infection

The HCV force of infection for PWID in each intervention state $j$ is denoted by $\beta_{j}$ and is given by:

$$\beta_{j}={\Phi_{j}^{HCV}\beta}_{inj}^{HCV}\frac{C_{-}+C_{+}}{T}$$

where

$$C_{-}=\sum_{i} \sum_{j} \sum_{k} {\Phi_{j}^{HCV}X}_{i,j,k}^{1,3}$$

$$C_{+}=\sum_{i} \sum_{j} \sum_{k} \sum_{m\geq2} {F\Phi}_{j}^{HCV}X_{i,j,k}^{m,3}$$

$$T=\sum_{i} \sum_{j} \sum_{k} \sum_{m} \sum_{n} \Phi_{j}^{HCV}X_{i,j,k}^{m,n}$$

where

- $\beta_{inj}^{HCV}$ is the HCV transmission rate for PWID who are not currently accessing OAT or NSP and are susceptible to HIV.
- $\Phi_{j}^{HCV}$ denotes the relative reduction in HCV transmission for injecting transmission if accessing OAT ($j=2$), NSP $(j=3,6)$ or both $\left( j=4 \right)$. Note that when not accessing OAT or NSP $(j=1,5)$ $\Phi_{j}^{HCV}=1$.
- $F$ is the relative increase in HCV transmissibility if PWID are HIV positive compared to HIV negative.

### Non-HIV related mortality and injecting cessation - $M_{i,j,k}^{m,n}$

These terms are concerned with cessation of injecting and non-HIV related mortality.

$$M_{i,j,k}^{m,n}=-\left( \mu_{i,1}+\mu_{2} \right)X_{i,j,k}^{m,n}$$

where

- $\mu_{i,1}$ is the non-HIV related mortality rate for gender $i$.
- $\mu_{2}$ denotes the rate of cessation of injecting

# Results

Supplementary Figure 3 shows the relative decrease in HIV incidence over time for different years. The immediate relative decrease is seen more among male PWID than female PWID – between 2019 and 2020 there is a 40.7% reduction in HIV incidence among male PWID compared to a 30.8% decrease in HIV incidence among female PWID. Over 2019-2022 the decrease in incidence has increased to 54.5% and 47.7% among male and female PWID, respectively, and over 2019-2025 59.5% and 54.7% decrease among male and female PWID, respectively.

***Supplementary Figure 3:*** *The relative decrease in HIV incidence over time from 2019. For each year on the axis the relative reduction in incidence from 2019 to that year was calculated. The figure shows for male PWID (blue line) and female PWID (orange line), respectively.*


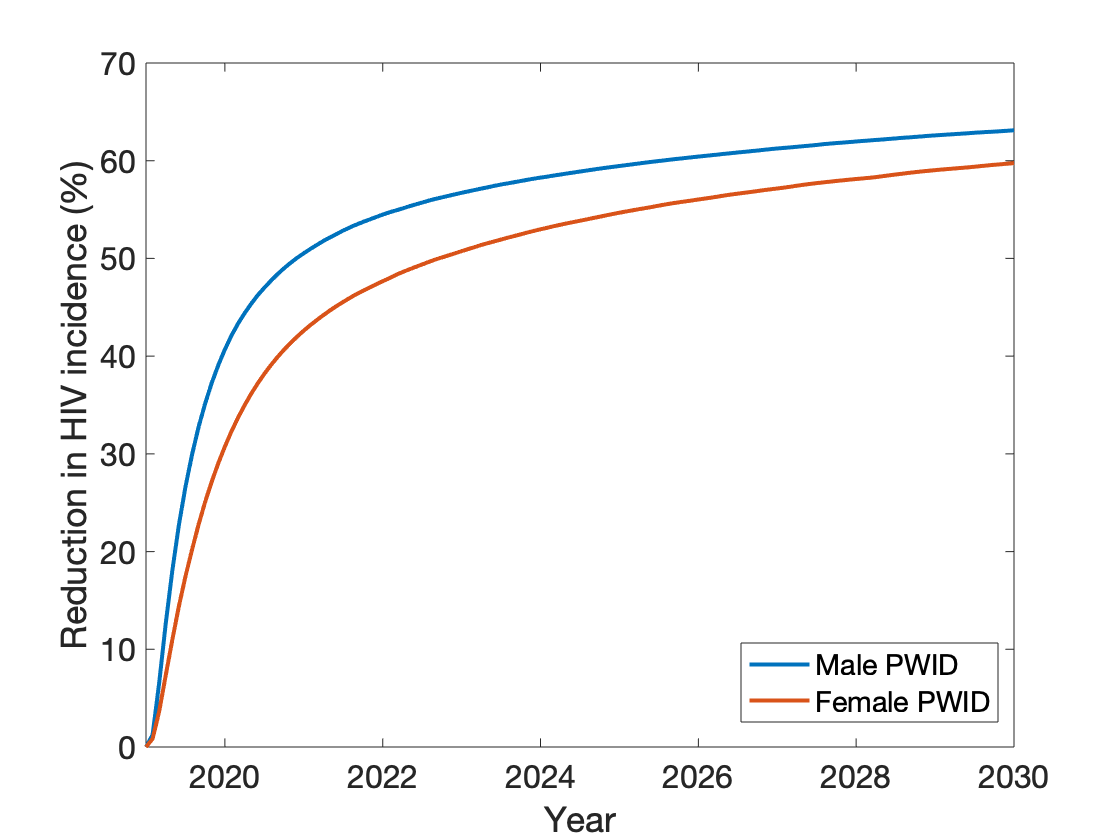


Supplementary Figure 4 shows the relative reduction in HIV incidence when assuming scaling up harm reduction, ART and different reductions in sexual transmission risk (25%, 50% and 75%). Full harm reduction, ART and interventions resulting in a 25% reduction in sexual transmission risk is likely to achieve a 74.6% (95%CrI:65.1–82.6%) reduction in incidence over 2019-2030, compared to an 86.9% (95%CrI:81.2–91.8%) reduction in incidence if sexual transmission is reduced by 75%.

***Supplementary Figure 4:*** *Box plot showing the relative change in HIV incidence among people who inject drugs (PWID) between 2019 and 2030 under different intervention scenarios. Note that boxes represent the median and 25^th^ to 75^th^ percentile range and whiskers represent 2.5^th^ to 97.5^th^ percentiles. Status quo is shown in blue shading while intervention scenarios are (orange shading) Full harm reduction (full HR; scaling up opioid agonist therapy (OAT) to 50% coverage and needle and syringe programmes (NSP) to 75% coverage), ART to high coverage and decrease sexual risk by 25%; (yellow shading) Full HR, ART to high coverage and decrease sexual risk by 30%; (purple shading) Full HR, ART to high coverage and decrease sexual risk by 75%.*


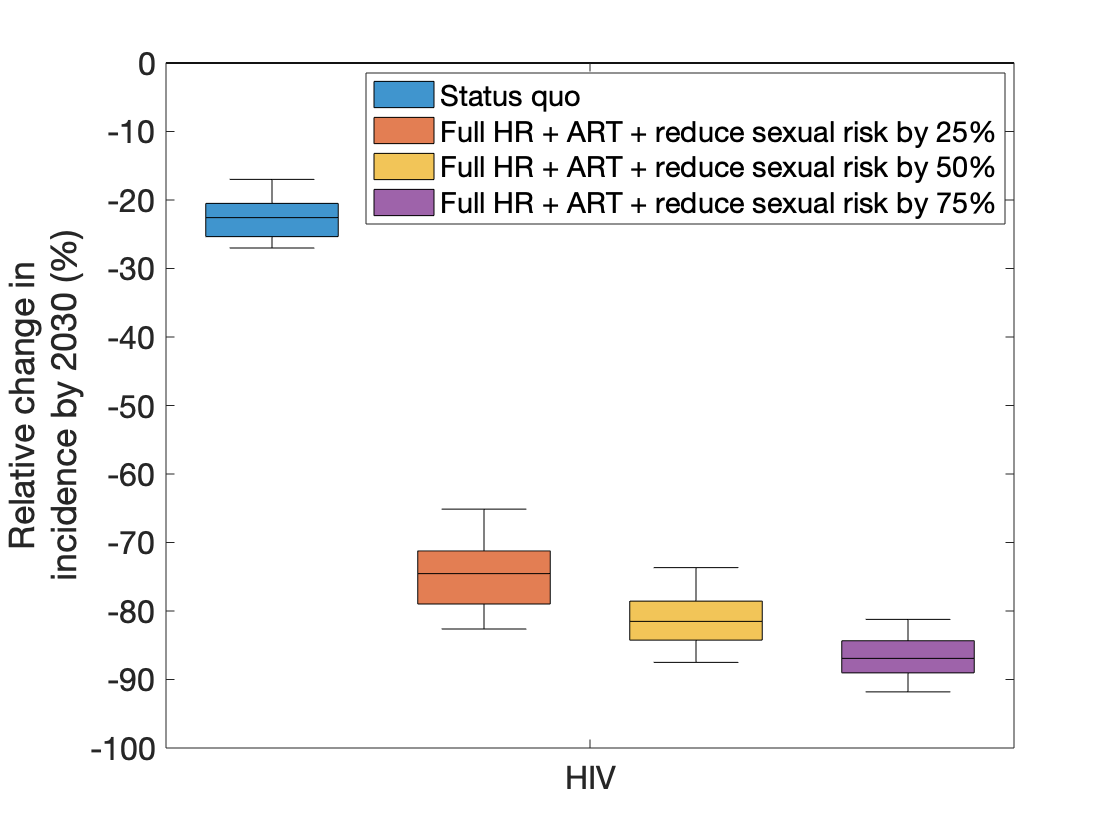


Supplementary Figure 5 shows the relative reduction in HIV incidence by gender for the different intervention scenarios. The impact of full HR, ART alone and full HR + ART have similar impact among males and females. Full HR alongside ART and interventions to reduce sexual risk by 75% have slightly greater impact among females than males.

***Supplementary Figure 5:*** *Box plot showing the relative reduction in HIV incidence among people who inject drugs (PWID) between 2019 and 2030 under different intervention scenarios. Note that boxes represent the median and 25^th^ to 75^th^ percentile range and whiskers represent 2.5^th^ and 97.5^th^ percentiles. Interventions scenarios by colour, with blue shading: scaling up opioid agonist therapy (OAT) to 50% coverage and needle and syringe programmes (NSP) to 75% coverage –denoted as full harm reduction (****Full HR****); orange shading: scaling-up antiretroviral therapy (ART) to 81% coverage with 90% of those on ART virally supressed; yellow shading: Full HR and ART to high coverage; purple shading: Full HR, ART to high coverage and decrease sexual risk by 75%.*

**
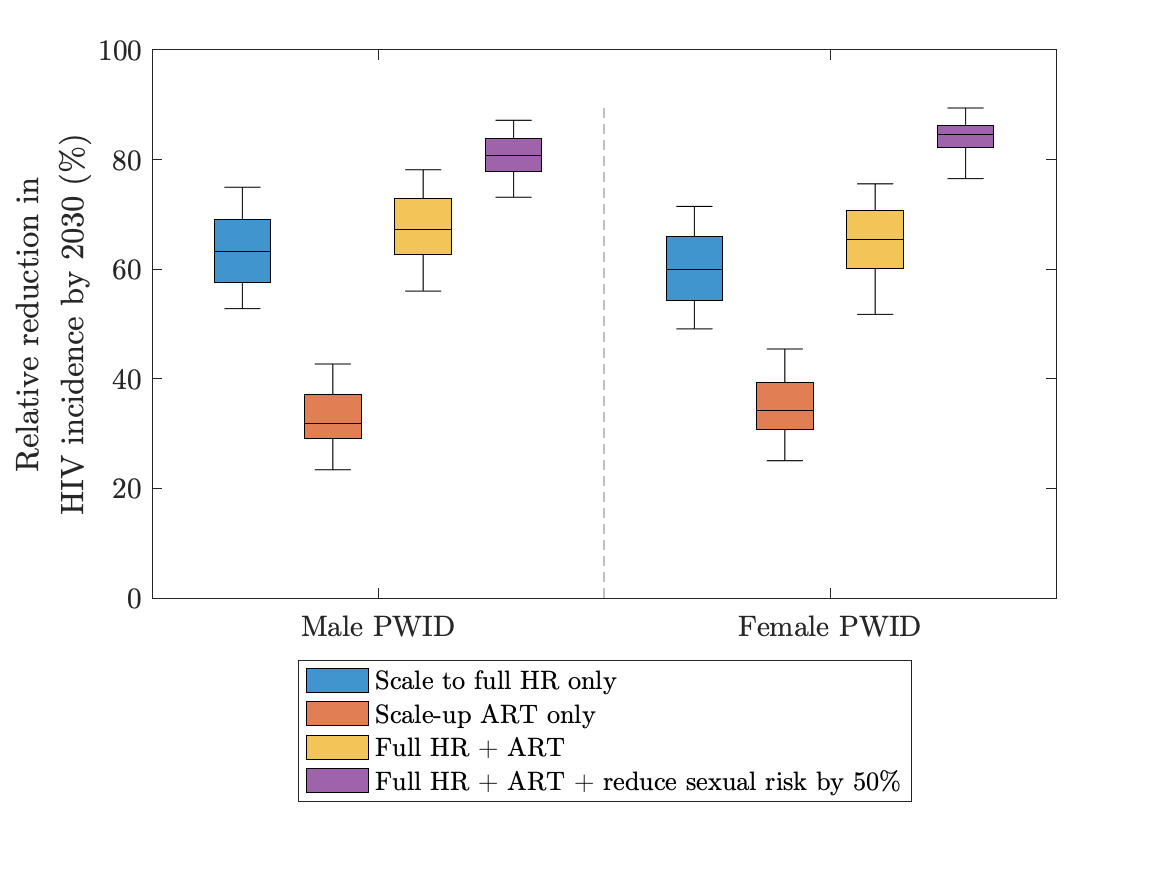
**

Supplementary Figure 6 shows the relative reduction in HCV incidence when assuming difference DAA treatment scenarios. The main text shows treating 10% of infected PWID annually, while Supplementary Figure 5 also shows the results for treating 5% or 15% of infected PWID annually, along or in combination with full harm reduction (50% OAT coverage and 50% NSP coverage).

***Supplementary Figure 6:*** *Box plot showing the relative change in HCV incidence among people who inject drugs (PWID) between 2019 and 2030 under different intervention scenarios. Note that boxes represent the median and 25^th^ to 75^th^ percentile range and whiskers represent 2.5^th^ to 97.5^th^ percentiles. Status quo is shown in blue shading while intervention scenarios are (orange shading) treat 5% of infected PWID with DAAs annually; ART to high coverage and decrease sexual risk by 25%; (yellow shading) treat 10% of infected PWID with DAAs annually; (purple shading) treat 15% of infected PWID with DAAs annually; (green sharing) full harm reduction (full HR; scaling up opioid agonist therapy (OAT) to 50% coverage and needle and syringe programmes (NSP) to 75% coverage) and treating 5% of infected PWID with DAAs annually; (pale blue shading) full HR and treating 10% of infected PWID with DAAs annually; (dark red shading) full HR and treating 15% of infected PWID with DAAs annually.*


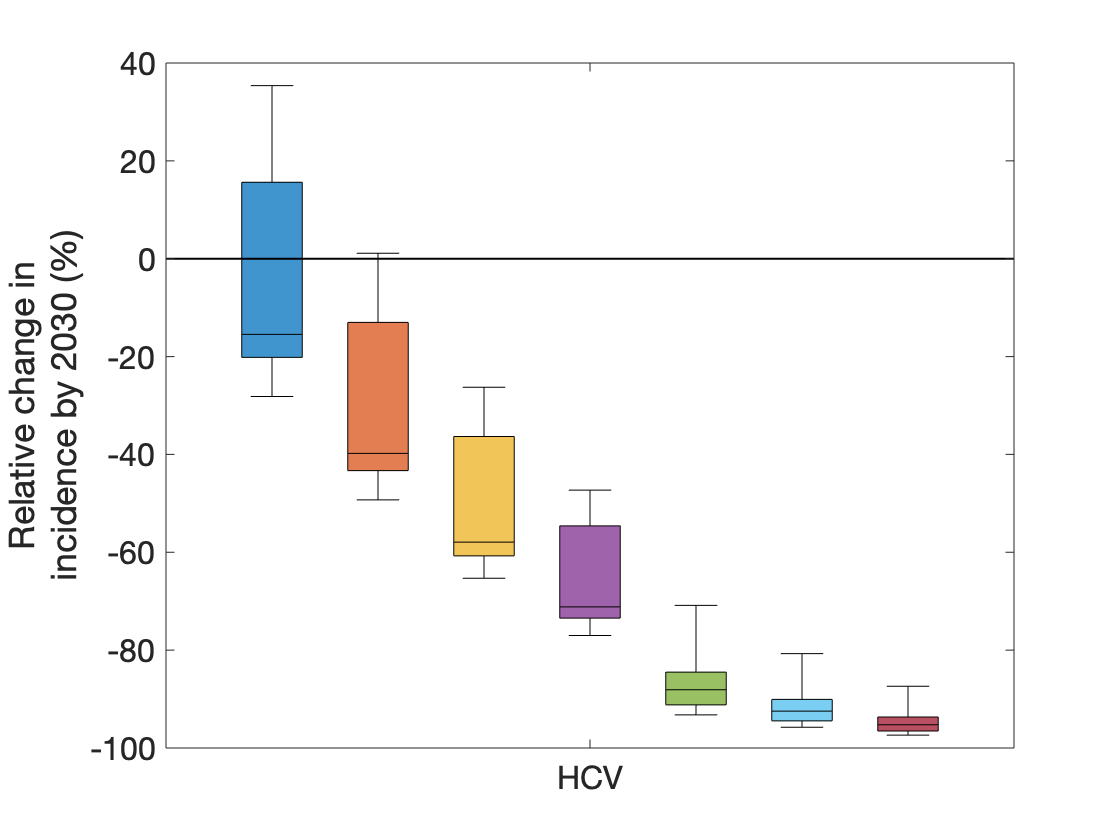


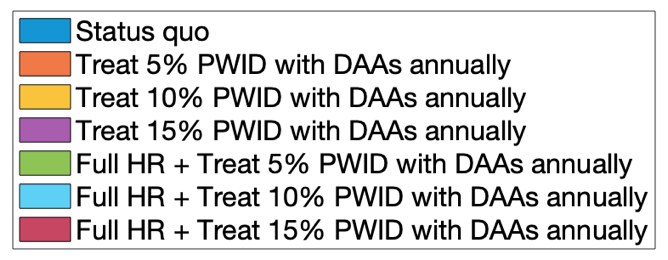


Supplementary figure 7 shows projections of HIV and HCV prevalence over time under the status quo scenario, and if the rate of recruitment onto ART is kept constant post 2011.

***Supplementary Figure 7:*** *Model projections for the* *(a) HIV prevalence amongst all PWID over time and (b) HCV prevalence amongst all PWID over time. The black line gives the median projections from 3,500 parameter sets, with 95% credibility intervals shown in red shading. The grey dot-dashed line shows the scenario where the rate of recruitment onto antiretroviral therapy is constant after 2011. Antiretroviral therapy started in 2004, OAT started in February 2011 and NSP started in March 2011. Grey circles and lines show the mean and 95% confidence interval of the data that the model was calibrated to, as given in Table 1. For (c), the blue line shows the range used for model calibration.*

(a) (b)


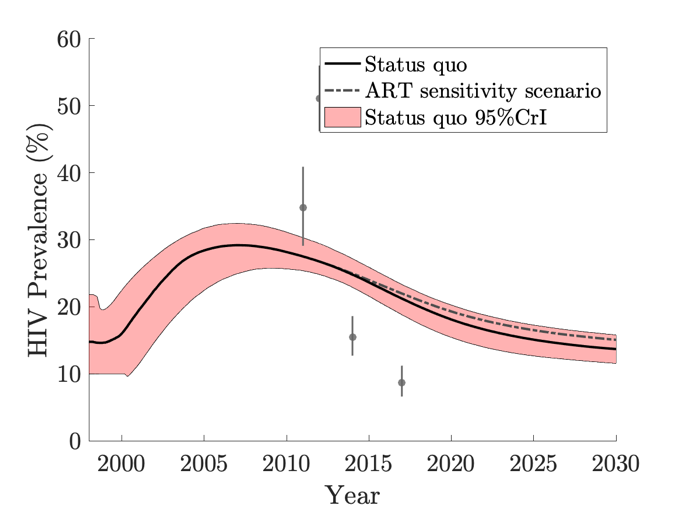

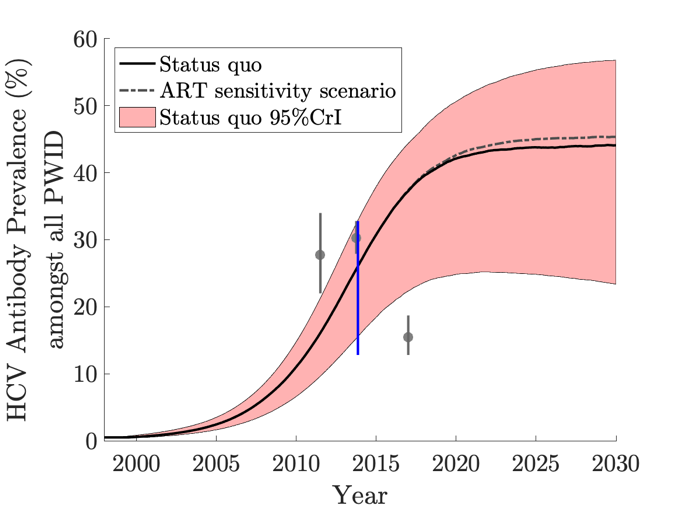


**Supplementary figure 8:** Box plot showing the relative change in HIV and HCV incidence among people who inject drugs (PWID) between 2019 and 2030 under different intervention scenarios. Note that boxes represent the median and 25^th^ to 75^th^ percentile range and whiskers represent 2.5^th^ to 97.5^th^ percentiles. Status quo is shown in blue shading while intervention scenarios are (orange shading) scaling up opioid agonist therapy (OAT) to 50% coverage and needle and syringe programmes (NSP) to 75% coverage – denoted as full harm reduction (Full HR); (yellow shading) scaling-up OAT to 50% coverage; (purple shading) Scaling-up NSP to 75% coverage.

ART refers to anti-retroviral therapy; OAT stands for opioid agonist therapy; NSP stands for needle and syringe programmes; HCV stands for hepatitis C virus; PWID stands for people who inject drugs. HR stands for harm reduction; DAA stands for direct acting antivirals

**
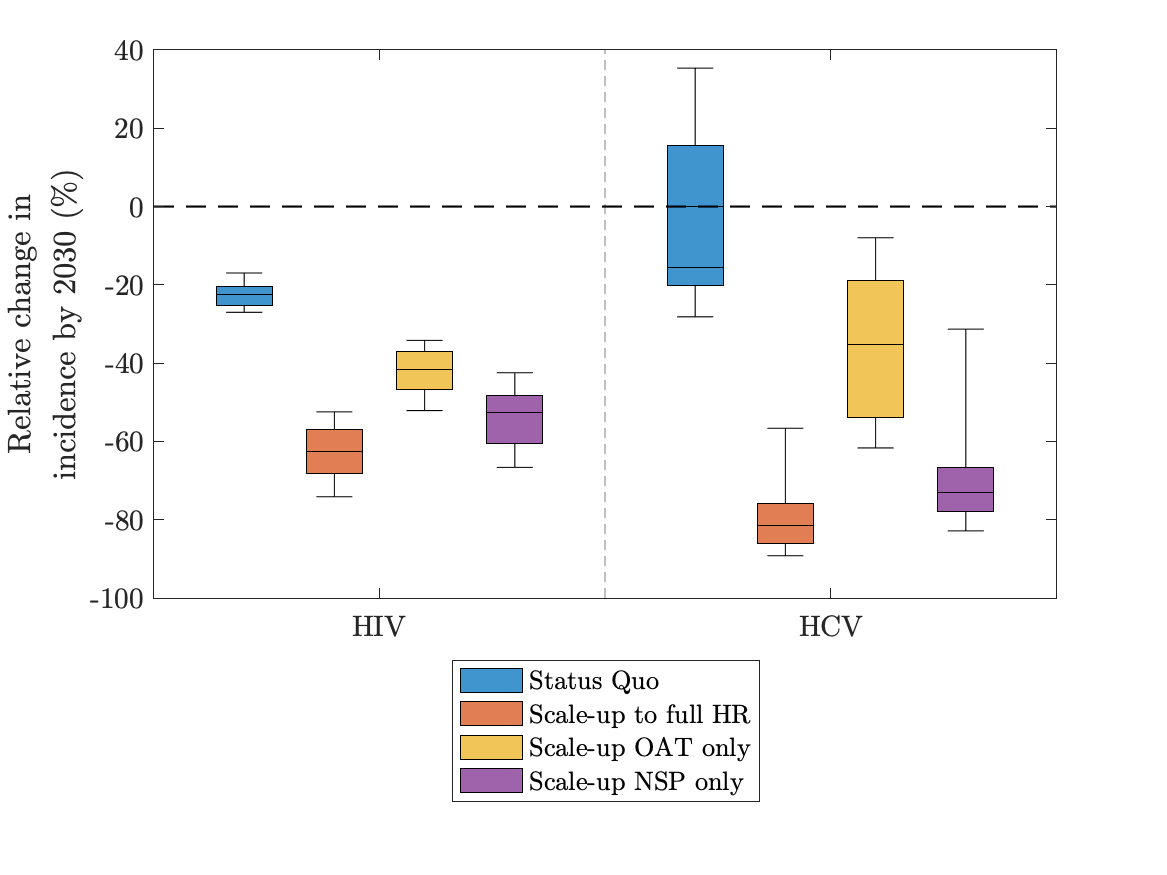
**

**Supplementary Figure 9:** Model projections for: (a) HIV prevalence and (b) HIV incidence. The black line gives the median projections for the status quo scenario, with 95% credibility intervals shown in grey shading. Median projections for intervention scenarios are: (red) scaling up opioid agonist therapy (OAT) to 50% coverage and needle and syringe programmes (NSP) to 75% coverage – denoted as full harm reduction (Full HR); (blue) scaling-up antiretroviral therapy (ART) to 81% coverage with 90% of those on ART virally supressed; (green) Full HR and ART to high coverage; (purple) Full HR, ART to high coverage and decrease sexual risk by 50%.

**
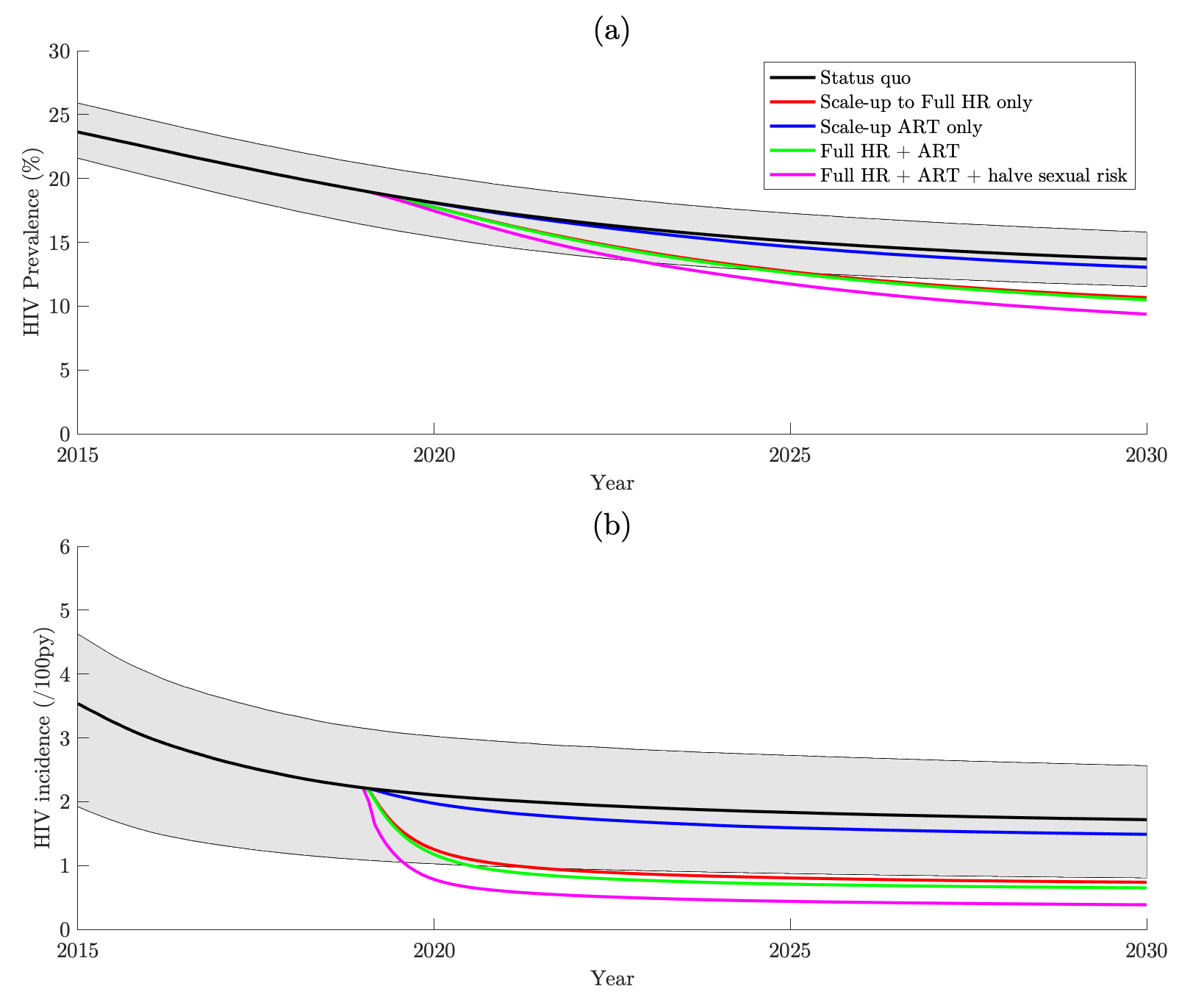
**

**Supplementary Figure 10:** Model projections for: (a) Chronic HCV prevalence and (b) HCV incidence. The black line gives the median projections for the status quo scenario, with 95% credibility intervals shown in gray shading. Median projections for intervention scenarios are: (red) scaling up opioid agonist therapy (OAT) to 50% coverage and needle and syringe programmes (NSP) to 75% coverage – denoted as full harm reduction (Full HR); (blue) treat 10% of HCV-infected PWID with direct-acting antivirals (DAAs) annually; (green) Full HR and treat 10% of HCV-infected PWID with DAAs annually.

**
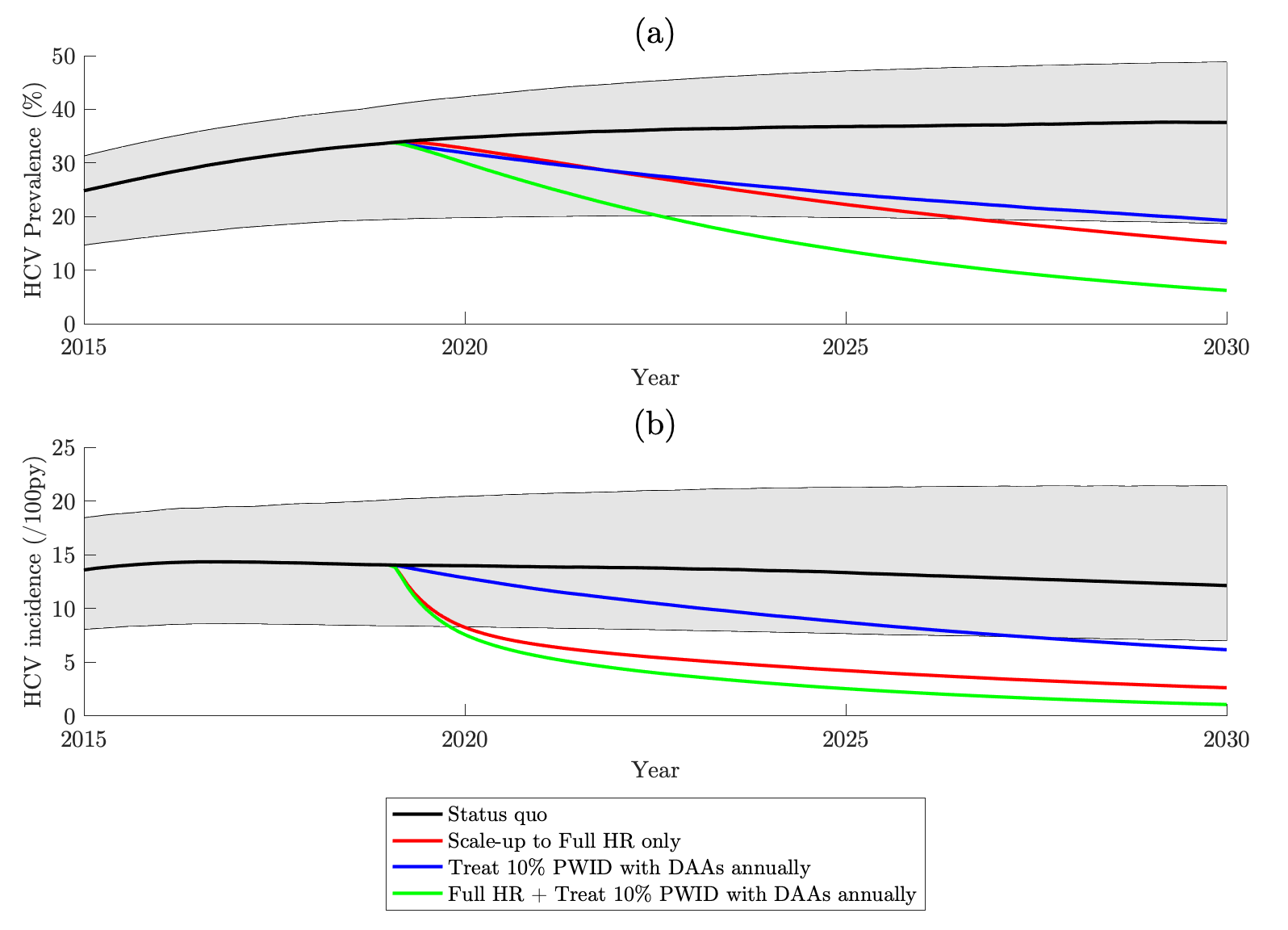
**

**Supplementary figure 11:** Box plot showing the relative decrease in HCV incidence among people who inject drugs (PWID) between 2019 and 2030 under different intervention scenarios. Note that boxes represent the median and 25^th^ to 75^th^ percentile range and whiskers represent 2.5^th^ to 97.5^th^ percentiles. Intervention scenarios are: (blue shading) scale-up OAT to 50% coverage and needle and syringe programmes (NSP) to 75% coverage – denoted as full harm reduction (Full HR); (red) treat 10% of HCV-infected PWID with direct-acting antivirals (DAAs) annually; (yellow) Full HR and treat 10% of HCV-infected PWID with DAAs annually. Boxes in the left-hand panel show results for all model runs; boxes in the middle panel show results for model runs which have decreasing HCV incidence in 2019; boxes in the right-hand panel show results for model runs which have increasing HCV incidence in 2019.

#
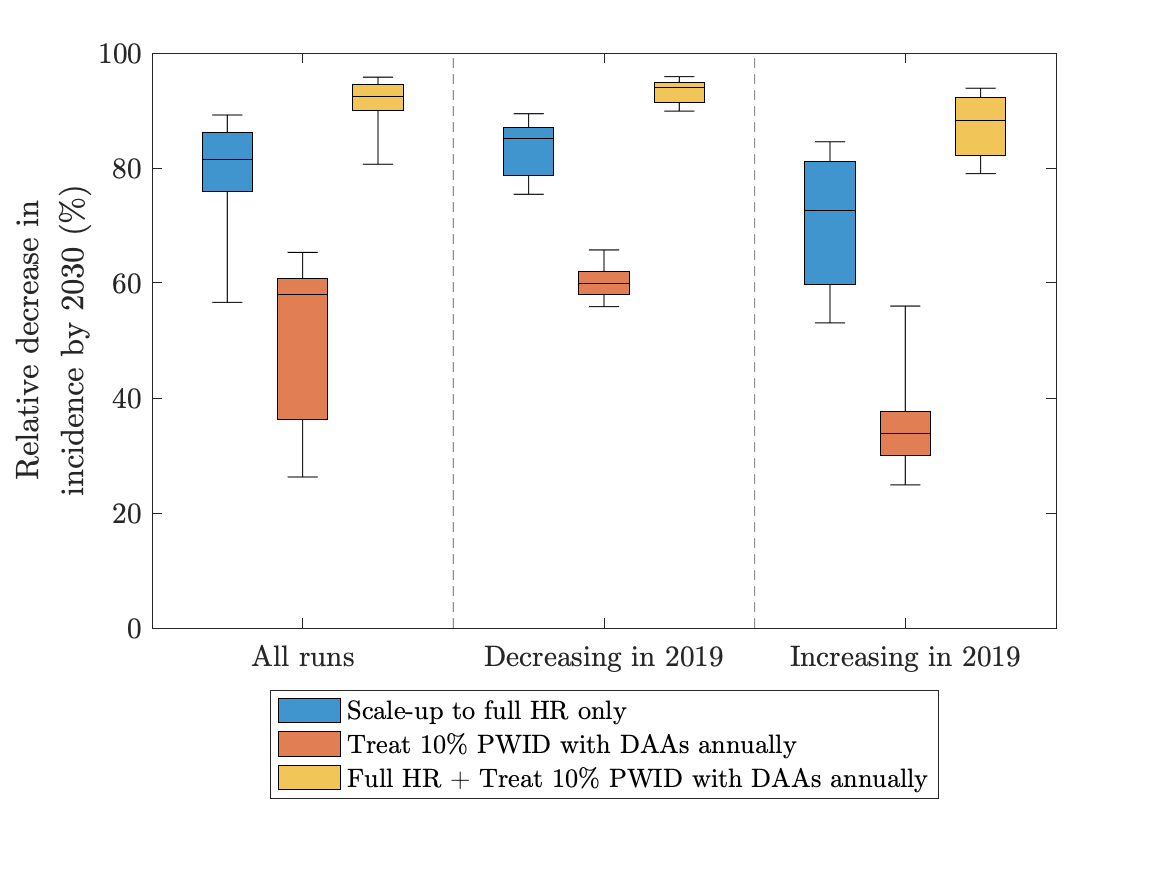


**Supplementary figure 12:** Results of sensitivity analysis assuming 25% assortative mixing by harm reduction status. Model projections for the HIV prevalence amongst (a) male PWID and (b) female PWID over time; (c) HCV antibody prevalence amongst all PWID. For each, the black line gives the median projections from 3,500 parameter sets, with 95% credibility intervals shown in red shading. Grey circles and lines show the mean and 95% confidence interval of the data that the model was calibrated to, as given in Table 1. For (c), the blue line shows the range used for model calibration.


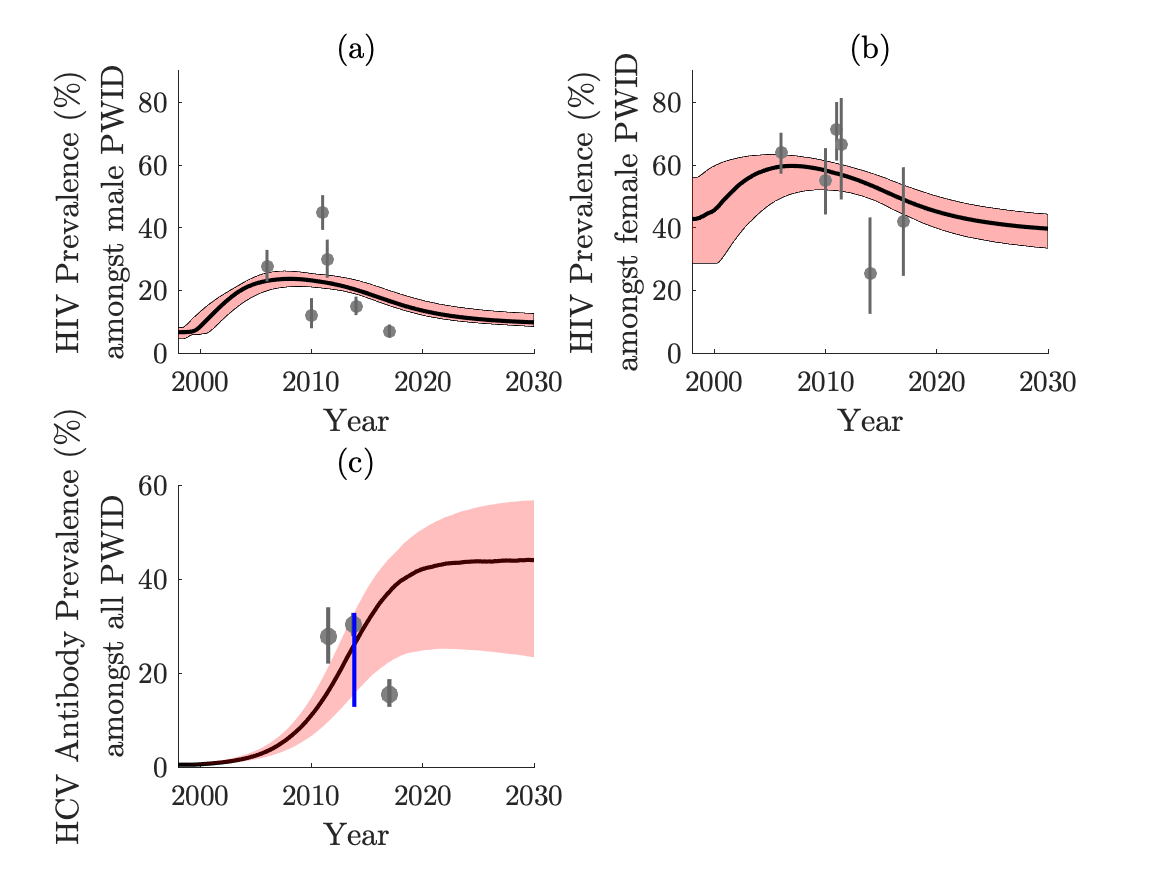


**Supplementary figure 13:** Results of sensitivity analysis assuming 50% assortative mixing by harm reduction status. Model projections for the HIV prevalence amongst (a) male PWID and (b) female PWID over time; (c) HCV antibody prevalence amongst all PWID. For each, the black line gives the median projections from 3,500 parameter sets, with 95% credibility intervals shown in red shading. Grey circles and lines show the mean and 95% confidence interval of the data that the model was calibrated to, as given in Table 1. For (c), the blue line shows the range used for model calibration.


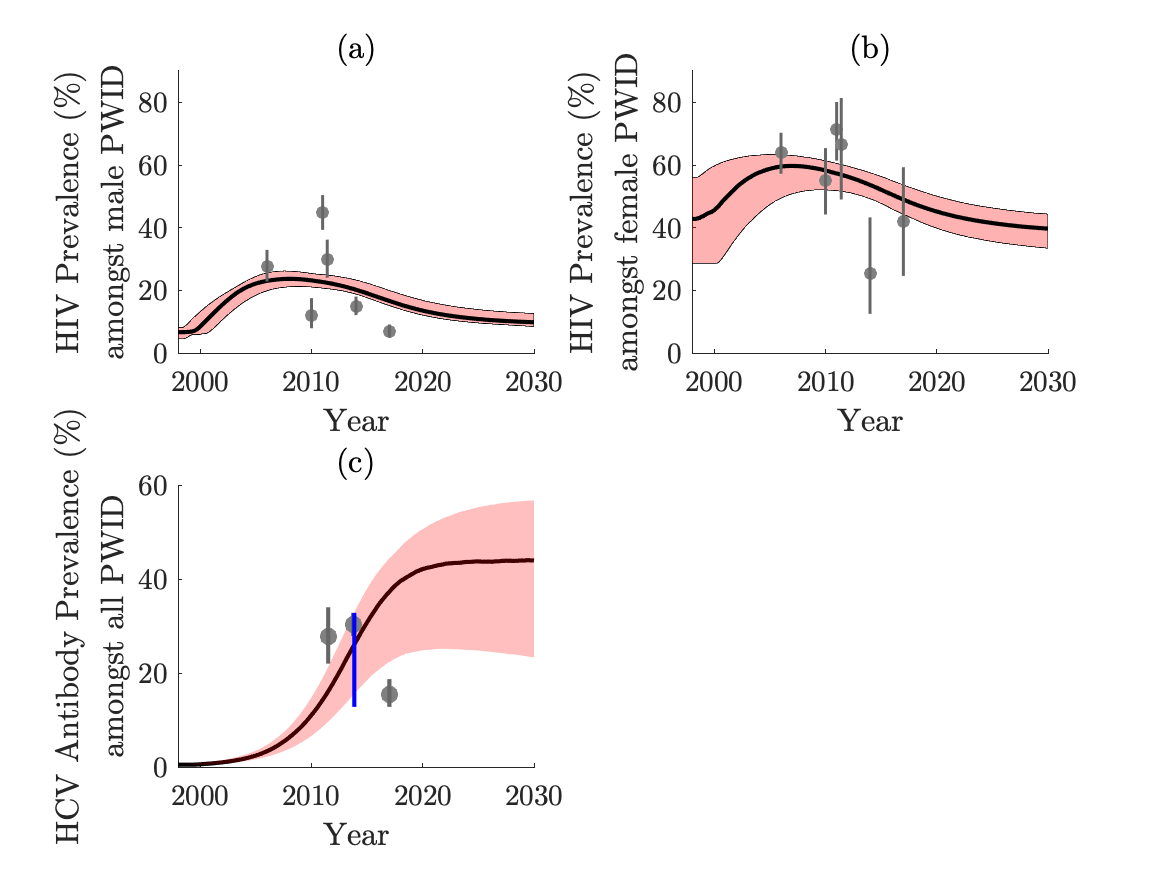


**Supplementary Table 4:** Relative change in HIV incidence among people who inject drugs (PWID) between 2019 and 2030 under different intervention scenarios for the baseline model fits, and sensitivity analyses which assume 25% or 50% assortative (like-with like) mixing by harm reduction status. Cells show the median value with 95%CrI presented in parentheses.

|  | Baseline | 25% assortative mixing | 50% assortative mixing |
| --- | --- | --- | --- |
| Status Quo | -22.57%  (-27.01 , -16.98) | -22.52%  (-26.99 , -16.96) | -22.47%  (-26.98 , -16.93) |
| Scale to full HR only | -62.61%  (-74.12 , -52.46) | -62.65%  (-74.18 , -52.5) | -62.71%  (-74.25 , -52.55) |
| Scale-up ART only | -32.45%  (-43.23 , -23.91) | -32.39%  (-43.23 , -23.92) | -32.33%  (-43.23 , -23.92) |
| Full HR + ART | -66.9%  (-77.57 , -55.58) | -66.94%  (-77.60, -55.64) | -66.98%  (-77.64 , -55.69) |
| Full HR + ART + reduce sexual risk by 50% | -81.53%  (-87.48 , -73.66) | -81.55%  (-87.5 , -73.68) | -81.56%  (-87.53 , -73.7) |

**Supplementary Table 5:** Relative change in HCV incidence among people who inject drugs (PWID) between 2019 and 2030 under different intervention scenarios for the baseline model fits, and sensitivity analyses which assume 25% or 50% assortative (like-with like) mixing by harm reduction status. Cells show the median value with 95%CrI presented in parentheses

|  | Baseline | 25% assortative mixing | 50% assortative mixing |
| --- | --- | --- | --- |
| Status Quo | -15.46%  (-28.16 , 35.38) | -15.69%  (-28.22 , 35.28) | -15.92%  (-28.3 , 35.16) |
| Scale to full HR only | -81.41%  (-89.21 , -56.63) | -81.56%  (-89.29 , -56.84) | -81.75%  (-89.35 , -57.07) |
| Scale-up ART only | -16.93%  (-30.53 , 34.51) | -17.15%  (-30.67 , 34.33) | -17.4%  (-30.75 , 34.18) |
| Full HR + ART | -81.39%  (-89.22 , -56.57) | -81.58%  (-89.31 , -56.77) | -81.75%  (-89.39 , -56.98) |
| Treat 10% PWID with DAAs annually | -57.92%  (-65.32 , -26.27) | -57.99%  (-65.36 , -26.33) | -58.09%  (-65.4 , -26.4) |
| Full HR + Treat 10% PWID with DAAs annually | -92.45%  (-95.79 , -80.66) | -92.51%  (-95.82 , -80.73) | -92.59%  (-95.84 , -80.81) |

# References

1. Bowring AL, van Gemert C, Dietze P, Toufik A, Stoove M. Assessment of risk practices and infectious disease among drug users in Temeke District, Dar es Salaam, Tanzania. Prepared for Médecins du Monde – France Melbourne: Centre for Population Health, Burnet Institute. 2011.

2. Ross MW, McCurdy SA, Kilonzo GP, Williams ML, Leshabari MT. Drug use careers and blood-borne pathogen risk behavior in male and female Tanzanian heroin injectors. Am J Trop Med Hyg. 2008;79(3):338-43.

3. Williams ML, McCurdy SA, Atkinson JS, Kilonzo GP, Leshabari MT, Ross MW. Differences in HIV risk behaviors by gender in a sample of Tanzanian injection drug users. Aids Behav. 2007;11(1):137-44.

4. Hollingsworth TD, Anderson RM, Fraser CJTJoid. HIV-1 transmission, by stage of infection. 2008;198(5):687-93.

5. Aspinall EJ, Nambiar D, Goldberg DJ, Hickman M, Weir A, Van Velzen E, et al. Are needle and syringe programmes associated with a reduction in HIV transmission among people who inject drugs: a systematic review and meta-analysis. Int J Epidemiol. 2014;43(1):235-48.

6. MacArthur G, Minozzi S, Martin N, Vickerman P, Deren S, Bruneau J. Evidence for the effectiveness of opioid substitution treatment in relation to HIV transmission in people who inject drugs: a systematic review and meta-analysis. BMJ. 2012;345:e5945.

7. Quinn TC, Wawer MJ, Sewankambo N, Serwadda D, Li C, Wabwire-Mangen F, et al. Viral load and heterosexual transmission of human immunodeficiency virus type 1. 2000;342(13):921-9.

8. Platt L, Minozzi S, Reed J, Vickerman P, Hagan H, French C, et al. Needle syringe programmes and opioid substitution therapy for preventing hepatitis C transmission in people who inject drugs. Cochrane Database Syst Rev. 2017;9:CD012021.

9. Fraser H, Zibbell J, Hoerger T, Hariri S, Vellozzi C, Martin NK, et al. Scaling up HCV prevention and treatment interventions in rural USA–model projections for tackling an increasing epidemic. Addiction. 2018;113(1):173-82.

10. Smith DJ, Jordan AE, Frank M, Hagan HJBid. Spontaneous viral clearance of hepatitis C virus (HCV) infection among people who inject drugs (PWID) and HIV-positive men who have sex with men (HIV+ MSM): a systematic review and meta-analysis. 2016;16(1):471.

11. Micallef JM, Kaldor JM, Dore GJ. Spontaneous viral clearance following acute hepatitis C infection: a systematic review of longitudinal studies. J Viral Hepat. 2006;13(1):34-41.

12. American Association for the Study of Liver Diseases, America IDSo. Recommendations for Testing, Managing, and Treating Hepatitis C. 2017;<http://www.hcvguidelines.org/contents> (Archived at <http://www.webcitation.org/6t6OPVwIZ> on 30 August 2017).

13. Low AJ, Mburu G, Welton NJ, May MT, Davies CF, French C, et al. Impact of opioid substitution therapy on antiretroviral therapy outcomes: a systematic review and meta-analysis. 2016;63(8):1094-104.

14. Ministry of Health CD, Gender, Elderly and Children, . Integrated Bio-Behavioral Surveillance Survey among People who Inject Drugs in Dar es Salaam, 2017. 2018.

15. Bowring AL, Luhmann N, Pont S, Debaulieu C, Derozier S, Asouab F, et al. An urgent need to scale-up injecting drug harm reduction services in Tanzania: prevalence of blood-borne viruses among drug users in Temeke District, Dar-es-Salaam, 2011. Int J Drug Policy. 2013;24(1):78-81.

16. Lambdin BH, Bruce RD, Chang O, Nyandindi C, Sabuni N, Zamudio-Haas S, et al. Identifying programmatic gaps: inequities in harm reduction service utilization among male and female drug users in Dar es Salaam, Tanzania. PLoS ONE. 2013;8(6):e67062.

17. Lambdin BH, Masao F, Chang O, Kaduri P, Mbwambo J, Magimba A, et al. Methadone treatment for HIV prevention-feasibility, retention, and predictors of attrition in Dar es Salaam, Tanzania: a retrospective cohort study. Clin Infect Dis. 2014;59(5):735-42.

18. Mohamed Z, Rwegasha J, Kim JU, Shimakawa Y, Poiteau L, Chevaliez S, et al. The hepatitis C cascade of care in people who inject drugs in Dar es Salaam, Tanzania. 2018.

19. Dutta A, Barker C, Makyao N. Consensus estimates on key population size and HIV prevalence in Tanzania. 2014. 2015.

20. PEPFAR. Tanzania Operational Plan Report FY 2013. Available from <https://www.state.gov/wp-content/uploads/2019/08/Tanzania-10.pdf>. 2013.

21. Mocroft A, Kirk O, Aldins P, Chies A, Blaxhult A, Chentsova N, et al. Loss to follow‐up in an international, multicentre observational study. 2008;9(5):261-9.

22. Toni T, Welch D, Strelkowa N, Ipsen A, Stumpf MPJJotRSI. Approximate Bayesian computation scheme for parameter inference and model selection in dynamical systems. 2009;6(31):187-202.

23. Williams ML, McCurdy SA, Bowen AM, Kilonzo GP, Atkinson JS, Ross MW, et al. HIV seroprevalence in a sample of Tanzanian intravenous drug users. AIDS Educ Prev. 2009;21(5):474-83.

24. Atkinson J, McCurdy S, Williams M, Mbwambo J, Kilonzo GJAjod, studies a. HIV risk behaviours, perceived severity of drug use problems, and prior treatment experience in a sample of young heroin injectors in Dar es Salaam, Tanzania. 2011;10(1).

25. Nyandindi C. HIV serostatus, hepatitic C and depression among injection drug users in Kinondoni municuipality, Dar es Salaam: Doctoral dissertation, Muhimbili University of Health and Allied Sciences; 2011.

26. Tran OC, Bruce RD, Masao F, Ubuguyu O, Sabuni N, Mbwambo J, et al. Implementation and Operational Research: Linkage to Care Among Methadone Clients Living With HIV in Dar es Salaam, Tanzania. Journal of acquired immune deficiency syndromes (1999). 2015;69(2):e43-8.

27. Quinn TC, Wawer MJ, Sewankambo N, Serwadda D, Li C, Wabwire-Mangen F, et al. Viral load and heterosexual transmission of human immunodeficiency virus type 1. Rakai Project Study Group. N Engl J Med. 2000;342(13):921-9.

28. Hughes JP, Baeten JM, Lingappa JR, Magaret AS, Wald A, de Bruyn G, et al. Determinants of per-coital-act HIV-1 infectivity among African HIV-1-serodiscordant couples. The Journal of infectious diseases. 2012;205(3):358-65.
